# Transcriptional control of tomato fruit development and ripening

**DOI:** 10.1093/jxb/eraf357

**Published:** 2025-08-08

**Authors:** Rufang Wang, Ruud A de Maagd

**Affiliations:** Institute of Facility Agriculture, Guangdong Academy of Agricultural Sciences, Guangzhou 510640, China; Bioscience, Wageningen University & Research, Wageningen 6708 PB, The Netherlands; University of California, Davis, USA

**Keywords:** Ethylene, fruit development, regulatory network, ripening, tomato, transcription factor

## Abstract

Tomato is one of the most consumed vegetable crops worldwide as its fruits are highly palatable and provide nutrition and vitamins. Scientifically, it is the model crop for fleshy fruit development and ripening studies, with a well-annotated genome and ample resources. Fruit development and ripening are complex processes regulated at different organizational levels, in which plant hormones (ethylene, auxin, gibberellin, and abscisic acid) and transcriptional, post-transcriptional, and epigenetic regulation form regulatory cascades or networks to function coordinately. Here, we review recent progress and address remaining questions in relation to the transcriptional regulation of tomato fruit development by transcription factors, as well as highlighting the links with the other regulatory components.

## Teaser

We review recent progress and address remaining questions in relation to tomato fruit development and ripening transcriptional regulation, and highlight future challenges.

## Introduction

Tomato (*Solanum lycopersicum*) belongs to the *Solanaceae* family (nightshades). With its fleshy fruits containing nutrients, minerals, and antioxidants that are important components of the human diet, tomato ranks among the most consumed vegetables in the world.

As in all angiosperms, tomato fruits develop from ovaries, where the ovary wall develops to become the fleshy, thick pericarp. The successful pollination and fertilization of a flower is the signal to start growth, which is characterized by a fast increase in size resulting from cell divisions followed by cell expansion. Seeds develop from ovules if fertilization is accomplished, but when seed set is impaired, the fruits remain small, as there is a positive correlation between the seed number and the final fruit size (Varga and Bruinsma, 1976). Several spontaneous mutations and transgenic approaches produce parthenocarpic fruits without fertilization, which can be important to ensure yield stability (Maupilé *et al*., 2024).

Fruit ripening is a complex process involving biochemical and physiological changes. Fruits can be divided into climacteric and non-climacteric groups based on whether there is a peak in respiration at the beginning of ripening, usually in concert with a burst of ethylene production. Tomato fruits are climacteric, and ripening results in changes in their colour, flavour, and texture.

Tomato is an excellent model plant for fleshy fruit development and climacteric ripening studies (Alexander and Grierson, 2002; Giovannoni, 2004) because it is an autogamous diploid and has a relatively simple and small genome (∼950 Mb), a range of well-characterized single gene mutants, and introgression lines. The tomato genome was first sequenced in 2012 (Sato *et al*., 2012), and improved assemblies and annotations have appeared regularly since then. In addition, the resequencing of genomes of tomato accessions and related wild species has resulted in a wealth of information (Aflitos *et al*., 2014; Lin *et al*., 2014). More recently, using more advanced sequencing technology, several high-quality genomes were assembled (Szymański *et al*., 2020; Su *et al*., 2021). Tomato pan-genomes (L. Gao *et al*., 2019) and a super-pangenome (N. Li *et al*., 2023) uncovered new genes and alleles. The *de novo* assemblies of the two ancestral tomato species, *Solanum pimpinellifolium* and *S. lycopersicum* var. *cerasiforme*, help understand tomato domestication and genome-scale breeding (Takei *et al*., 2021). Long-read sequencing of 100 tomato genomes uncovered 238 490 structural variants that may affect gene expression (Alonge *et al*., 2020). Additionally, the relative ease of stable plant transformation and distinct ripening phenotypes make tomato an outstanding model for fruit ripening and gene function studies. Furthermore, decades of work have been undertaken on the biochemical changes underlying processes such as softening, colour changes, and the overall regulation of ripening.

A previous review from our laboratory summarized the knowledge of the transcriptional regulation of tomato fruit development and ripening at the time (Karlova *et al*., 2014). Since then, research has not only increased the known number of genes involved, not least through advances in targeted mutagenesis, but also overturned some of the assumptions about gene function resulting from the study of spontaneous mutants. This review will address recent findings and discoveries related to the transcriptional regulation of tomato fruit development and ripening, as well as briefly outline the effects of epigenetic regulation (Box 1), post-transcriptional regulation (Box 2), and structural variation (Box 3) on gene expression.

Box 1. Epigenetic regulation of gene expression Epigenetic modifications regulate gene expression without changes in the primary DNA sequence. They comprise DNA cytosine methylation or histone protein (de)methylation and (de)acetylation, which are often heritable and stable between generations. They modify chromatin structure to become more ‘open’ and accessible, or more ‘closed’ and restricted, for regulatory (TF) protein binding, leading to gene expression or silencing (Liu *et al*., 2010; Zhang *et al*., 2010; H. Zhang *et al*., 2018).

There is a global decrease of cytosine methylation (∼30%) in the pericarp during tomato fruit ripening (Teyssier *et al*., 2008), and most of the demethylation occurs in gene promoters (Lang *et al*., 2017). More evidence that DNA demethylation plays an active role in tomato ripening (Liu *et al*., 2015) came from a study in which the inactivation of the DNA demethylase gene *DEMETER-LIKE 2* (*SlDML2*) resulted in genome-wide DNA hypermethylation and the inhibition of fruit ripening (Lang *et al*., 2017). *DML2* expression is regulated by *NOR*, establishing its place in the ripening regulatory network shown in Fig. 2 (Gao *et al*., 2022).

The spontaneous epigenetic *Cnr* mutation has profound effects on ripening but also affects pre-ripening development (R. Wang *et al*., 2020), yet is distinctly different (dominant, epistatic) from *nor* and *rin*. It is characterized by hypermethylation of a short DNA region upstream of the TF gene *SPL-CNR*, inhibiting its ripening-related cytosine demethylation and reducing its expression. Hence, it was long assumed, understandably, that the phenotype reflected the master regulator function of the nearby TF gene. Only after CRISPR/Cas knockout of the gene revealed very minor effects on ripening was it realized that the epigenetic mutation has a much more profound and genome-wide effect on DNA methylation and gene expression. For example, the *Cnr* mutation prevents the binding of MADS-RIN to the promoters of many (if not all) of its targets, such as *ACS2*, *ACS4*, *RIN*, *NOR*, *FUL1*, and others (Martel *et al*., 2011). *MADS-RIN* and *FUL1* are silenced in the *dml2* mutant, the *rin/ful1* double mutant mimics the ripening phenotype of *dml2*, and *dml2* prevents the binding of RIN to its targets *in vivo*. Expression of *MADS-RIN* from a methylation-insensitive promoter restores the activation of many, although not all, MADS-RIN target genes, indicating that *dml2* blocks ripening through the inhibition of both expression and the binding of MADS-RIN to its targets (Niu *et al*., 2025).

A more recent study reported that RNA methylation is also involved in regulating tomato fruit ripening, suggesting a new layer of epigenetic control over fruit ripening (Zhou *et al*., 2019).

DNA cytosine demethylation is often the first step in chromatin remodelling, which is often followed by modification of the histones that comprise the nucleosomes, resulting in less compact chromatin that allows access by TFs (Tang *et al*., 2020). Indeed, it has been demonstrated that the promoters of many essential ripening genes become DNase I hypersensitive, a marker of accessibility, concurrent with a decrease in the marker of gene inactivity, H3K27me3 (Lü *et al*., 2018). One candidate for removing this inhibitory mark is the histone demethylase JUMONJI 6 (JMJ6), whose overexpression promotes ripening and the expression of many ripening-related genes (Li *et al*., 2020).

Box 2. Post-transcriptional regulation of gene expression **MicroRNA regulation**

In eukaryotes, only approximately 1–2% of the transcriptome encodes proteins (Birney *et al*., 2007), suggesting a large potential of additional roles for non-coding RNAs.

MicroRNAs are one category of non-coding RNAs. They are 20–22 nt in length and have been found in plants, animals, and some viruses. They function post-transcriptionally by binding to complementary sequences of mRNA of their target genes and subsequently cleave the mRNA or remain bound and inhibit its translation.

Many targets of microRNAs are TF-encoding genes. In tomato, Moxon *et al*. (2008) identified microRNAs by deep sequencing and showed that miR156/157 regulates *SPL-CNR in vivo*, and Ferreira e Silva *et al*. (2014) demonstrated that miR156 controls ovary and fruit development by being involved in the maintenance of the meristematic state of the ovary. Karlova *et al*. (2013), using degradome analysis, identified 119 target genes of microRNAs in different stages of fruit development, demonstrating the target cleavage activity of microRNAs in tomato fruits. In addition, they showed that the transcript cleavage of *SPL-CNR* by miR156/157 and *AP2a* by miR172 increased (30% for *SPL-CNR* and 48% for *AP2a*) at the breaker stage, indicating that they probably play roles in the regulation of tomato fruit ripening (Karlova *et al*., 2013). MicroRNAs regulate the expression of TF-encoding genes; conversely, TFs can also regulate their production, for example, MADS-RIN has a potential regulatory function on microRNA accumulation (Gao *et al*., 2015). MiR319 is involved in the regulation of tomato fruit shape by repressing the activity of SlTCP2/LANCEOLATE (Carvalho *et al*., 2025).

Long non-coding RNAs (lncRNAs) are non-protein-encoding transcripts that are longer than 200 nt; they regulate gene expression by histone modification, DNA methylation, and chromatin remodelling (Heo *et al*., 2013). They function in all plant developmental processes and play a crucial role during tomato fruit development and ripening (Sun and Xiao, 2015). LncRNA1459 (Li *et al*., 2018) and lncRNA1840 (Zhu *et al*., 2024) have been identified and proven, by CRISPR/Cas function studies, to be involved in ripening regulation. On the other hand, some lncRNAs are targets of ripening-related TFs; for example, 187 lncRNAs were identified as direct targets of RIN (Yu *et al*., 2019).

Box 3. Structural variations affecting transcription Structural variations (SVs) include large DNA insertions, deletions, duplications, and chromosomal rearrangements, affecting gene expression in plant developmental processes (Lye and Purugganan, 2019). With the sequencing of 100 tomato accessions, a pan-SV genome was established, and 238 490 structural variants were detected (Alonge *et al*., 2020). A specific type of SVs is related to transposable elements (TEs), leading to TE insertion polymorphisms (TIPS), which in some cases lead to the activation or inactivation of nearby gene transcription (Domínguez *et al*., 2020). More recently, with the emergence of more comprehensive pangenomes and super-pangenomes to detect differences in SV between wild and cultivated tomato species, more genomic diversity has been found, providing insights into tomato breeding (R. Wang *et al*., 2020; N. Li *et al*., 2023).

**Promoter deletions or insertions that affect gene expression**

TFs regulate gene expression only when bound to their specific binding sites in a gene, often in its 5′ promoter, so the presence of binding sites also determines the transcriptional regulation. TF binding sites belong to *cis*-regulatory elements (CREs), which are (often) non-coding DNA sequences that regulate the expression of nearby genes. *Cis*-regulation also comprises enhancers and silencers in other parts of the gene, such as introns, 3′ regions, or even exons, or far away from the regulated gene. Since multiple CREs regulate the same gene, including inhibitory ones, modifications of some CREs may not result in complete gene knockout but rather alter gene expression, so their effects are not ‘on’ or ‘off’, but ‘more’ or ‘less’. Mutations in CREs that cause *cis*-regulatory variations were frequent and often essential during plant domestication, and their effect is milder than that of mutations in the coding sequence of a gene that result in a change in the protein structure (Wittkopp and Kalay, 2011; Swinnen *et al*., 2016). A 3 bp InDel in the promoter of *Al-ACTIVATED MALATE TRANSPORTER9*, a key gene in malate synthesis and degradation, was selected during tomato domestication, which determines the fruit malate contents to improve aluminium tolerance (Ye *et al*., 2017). Other examples are the already mentioned *fas* allele, a genomic fragment inversion (∼294 kb) in the promoter region of *CLV3*, as well as the *lc* allele, which disrupts a negative-regulatory *cis*-element of the tomato *WUSCHEL* (*WUS*) gene (Huang and van der Knaap, 2011; Muños *et al*., 2011). Similarly, the ‘All-flesh’ fruit varieties described in this review contain a deletion in the promoter of *MBP3*, lowering its expression. Alonge *et al*. (2020) identified five different haplotypes of the *NON-SMOKY GLYCOSYLTRANSFERASE1* (*NSGT1*) and *NSGT2* paralogous gene locus, responsible for variation in guaiacol and methyl salicylate levels, varying in gene copy number (see below) and genomic deletions.

**Gene copy number effects**

Copy number variation (CNV) is a specific SV that modifies gene expression by directly affecting the gene copy number and is an important source of variation (Lye and Purugganan, 2019).

*SUN* is a major gene controlling tomato fruit elongation (resulting in round or elongated fruits). Its spontaneous mutant locus *sun* is caused by a 24.7 kb gene duplication mediated by the retrotransposon *Rider*, resulting in higher *SUN* expression, leading to a more elongated fruit shape (Xiao *et al*., 2008). The cytochrome P450 gene *KLUH* underlies the fruit weight locus *fw3*.2 and its expression positively affects tomato fruit weight (Chakrabarti *et al*., 2013). A tandem duplication of 50 kb at the *fw3*.2 locus, resulting in two copies of *KLUH*, led to higher expression and heavier fruits (Alonge *et al*., 2020).

## Transcriptional regulation of tomato fruit development

Transcription factors (TFs) are proteins that bind to specific regions of a gene, commonly the gene’s promoter, to activate or repress the transcription of that gene (Latchman, 1997). They are categorized in families according to their conserved domains, which are often involved in DNA binding. There are 1845 putative TFs in the tomato genome belonging to 58 TF families (Li *et al*., 2021).

As stated above, fruit development starts with the growth of the ovary after pollination, and fertilization causes the release of an inhibitory mechanism that blocks auxin action (Weijers and Wagner, 2016). IAA/AUX proteins recruit TOPLESS (TPL) to form a complex that functions as a repressor of AUXIN RESPONSE FACTOR (ARF)-activated transcription at low auxin levels, whereas this repression is released when auxin levels increase (Szemenyei *et al*., 2008; Lavy and Estelle, 2016). Although they initially suppress fruit set in the unfertilized ovary, four ARFs promote later fruit growth together with IAA9 (Hu *et al*., 2023).

Gibberellin (GA) and auxin signalling are important in fertilization, in which the GRAS [(GAI (gibberellic acid insensitive), RGA (repressor of GAI), and SCR (scarecrow)] TF DELLA (aspartic acid-glutamic acid-leucine-leucine-alanine) and ARFs play key roles (Huang *et al*., 2015; Hu *et al*., 2018). After fertilization, the auxin signal produced by seeds starts to stimulate the production of other hormones. Several members of the TF ARF family, whose expression increases after pollination, together control the IAA signalling pathway (Zhang *et al*., 2015; Yuan *et al*., 2018). GA is essential for normal fruit growth and its production is activated by IAA. The degradation of DELLA, which functions as a repressor of GA signalling, is required for fruit growth (Livne *et al*., 2015).

Pericarp identity and shape are, in large part, determined at the ovary stage and carried over to the developing fruit. Pericarp identity is co-determined by the MADS-domain (MCM1, AGAMOUS, DEFICIENS, and SRF) TFs TOMATO AGAMOUS 1 (TAG1) and TOMATO AGAMOUS-LIKE 1 (TAGL1), which are orthologs of Arabidopsis AG and SHATTERPROOF 1/2, respectively (Pnueli *et al*., 1994; Vrebalov *et al*., 2009; Pan *et al*., 2010). Like their Arabidopsis orthologs, C-type homeotic genes regulate aspects of carpel and ovule identity, and their mutation impacts on fruit growth and later ripening through their effect on organ identity (Gimenez *et al*., 2016). *TAG1* and *TAGL1* can thus be considered positive regulators of carpel identity, and at least the ectopic expression of *TAGL1* causes sepals to develop like carpels, including swelling and lycopene accumulation. The homeodomain-leucine zipper (HD-Zip) homeobox TF LeHB-1 may have a similar function, as its ectopic expression from a viral vector leads to the same phenotype (Lin *et al*., 2008).

The fruits of the wild tomato ancestor *S. pimpinellifolium* are small, weighing only ∼1 g and containing two locules. By contrast, cultivated tomato fruits can weigh 500 g and have more than 10 locules (Rodríguez-Leal *et al*., 2017). Eventual fruit size is, in large part, determined in the ovary, in particular by locule number. The ovary of a typical wild tomato or related species consists of two fused carpels. Variations in the transcriptional regulation of the floral meristem size, selected during domestication and breeding, often result in greater carpel number and, hence, larger fruit size (Tanksley, 2004). The TFs LOCULE NUMBER (LC) and EXCESSIVE NUMBER OF FLORAL ORGANS (ENO) function in a meristem regulatory network with similarity to the CLAVATA (CLV)–WUSCHEL (WUS)–AGAMOUS (AG) network in Arabidopsis (Xu *et al*., 2015). *LC* is the tomato ortholog of *WUS*, which is down-regulated by AG through its binding downstream of the coding sequence (Muños *et al*., 2011). In the *lc* mutant the presumed binding site is interrupted, leading to higher expression of *LC*/tomato *WUS* and a modest increase in carpel number and fruit size. *WUS* expression, which governs meristem size, is negatively controlled by the CLV2/CLV3 pathway. In the tomato *fasciated* (*fas*) mutant, *CLV3* expression is reduced by a genomic fragment inversion affecting its promoter (Xu *et al*., 2015). Reduced inhibition of *LC* due to lower CLV3 activity causes a strong increase in meristem size, with fasciated flowers and fruits and a large increase in fruit size (Cong *et al*., 2008). *LC* expression and carpel number are also increased by knocking out *ENO*, another inhibitor of *LC/WUS* expression, resulting in fasciated flowers and fruits (Fernández-Lozano *et al*., 2015; Yuste-Lisbona *et al*., 2020). The TFs ASYMMETRIC LEAVES 2 (SlAS2) and ASYMMETRIC LEAVES 2-LIKE (SlAS2L) belong to the LATERAL ORGAN BOUNDARIES (LOB) family, and are orthologs of Arabidopsis AS2, which regulates leaf polarity in conjunction with AS1 (Machida *et al*., 2015). These are also expressed in the ovary wall at anthesis and could thus also be considered to be co-determining pericarp identity. They are essential for tomato fruit growth, functioning in a partially redundant manner to regulate cell layer numbers and cell area in the pericarp, with knockout leading to smaller fruits. Their mutation resulted in the up-regulation of *PIN-FORMED 3* (*PIN3*) and the down-regulation of *GRETCHEN HAGEN 3* (*GH3*), which are involved in auxin transport and signalling, respectively, thus linking auxin and fruit growth (Dong *et al*., 2023).

*SUN* and *OVATE* control fruit shape, are both expressed early in flower and fruit development, and lead to an elongated fruit shape. *SUN* encodes a positive growth regulator, whereas *OVATE* encodes a negative one (Liu *et al*., 2002; Xiao *et al*., 2008). A Brassinazole Resistant 1 (BZR1)-like TF, BZR1.7, was found to regulate fruit elongation through promoting *SUN* expression (Yu *et al*., 2022). Besides, loss of function of the C2H2-type zinc finger TF POINTED TIP (PT) leads to fruit with a pointed tip via up-regulation of the expression of the MADS-domain TF gene *FRUITFULL2* (*FUL2*) (Song *et al*., 2022). The *fs8.1* (*fruit shape chr 8.1*) mutation in the GT-2 clade of trihelix TFs makes round fruits change to elongated through over-proliferation of the ovary wall cells, improving their crush resistance without changing the pericarp texture (Zhu *et al*., 2023).

There are two partially overlapping fruit growth phases defined after fruit set, the cell division phase and the cell expansion phase, and growth practically ceases from the onset of ripening (Gillaspy *et al*., 1993). In the cell division phase of approximately 7–10 d (Mapelli and National, 1978), the cell number increases, resulting in some fruit growth. Most of the increase in fruit size occurs in the cell expansion phase. During this phase there is an increase in cell volume with little additional cell division in the mesocarp (the parenchymatous central part of the former ovary wall, the pericarp) but continued division in the exocarp (resulting in smaller cells) until the mature green (MG) stage, after which cell volume barely changes. Cell expansion is accompanied by endoreduplication, in which the cell cycle and DNA synthesis continue to function but without them leading to mitosis. Thus, mesocarp cells reach ploidy levels of up to 256C (Bergervoet *et al*., 1996). This expansion is even more pronounced in the locular tissue, which, after initial growth to form parenchymatous tissue, liquefies to form the gel within the locules, enclosing the developing seeds. The D-class MADS-box gene *MBP3* is an essential regulator of this gel formation by controlling cell cycle and cell expansion genes in early fruit development. Natural variation in the gene’s promoter leads to reduced expression and consequent inhibition of gel formation and firmer fruits in varieties with the ‘All-flesh’ phenotype (Huang *et al*., 2021), indicating that pericarp and internal tissue development involve different trajectories and regulatory mechanisms.

Finally, four KNOX TFs, TKN1–4, were found to be specifically expressed in the tomato ovary wall at the early developmental stage (Zhang *et al*., 2016), among which TKN3 functions after fertilization to control cell expansion (Sun *et al*., 2025).

## Transcriptional regulation of ripening

Fruit ripening involves changes in the biochemistry and physiology of the fruit, resulting in altered colour (due to the production of carotenoids, particularly lycopene), flavour, and texture (softening). The initiation of ripening is generally defined as the breaker stage, when the first colour change at the blossom end can be observed (although colour change inside the fruit may occur earlier) and the auxin level has increased, as seeds are mature at the MG stage (Gillaspy *et al*., 1993). It takes approximately 35–50 d for a tomato to reach the ripening stage and an additional 5–10 d to become fully red and ripe. Although most studies of ripening regulation have focused on the pericarp, detailed transcriptomic analysis of pre-ripening stages separated into locular gel and pericarp indicated that extensive reprogramming occurs in the locular gel before it does in the pericarp (Chirinos *et al*., 2023). This indicates that ripening progresses from the inside outwards.

**Fig. 1.**
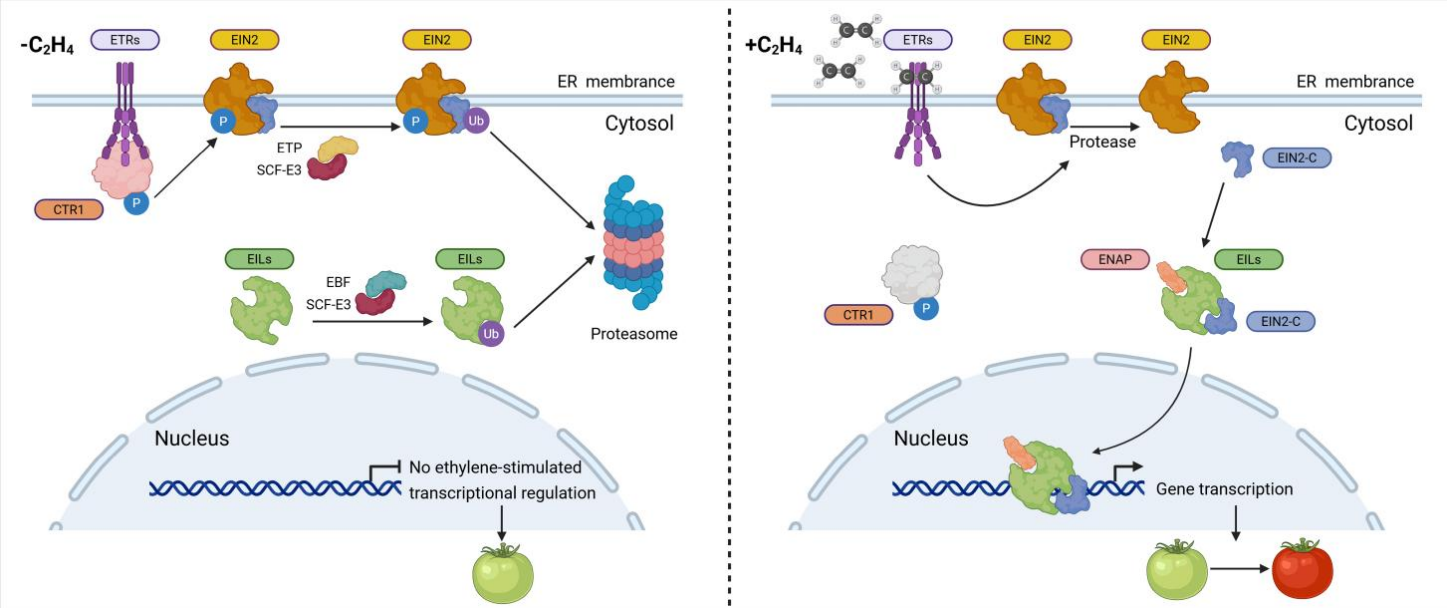
Ethylene signalling. Ethylene (C_2_H_4_) is perceived by ethylene receptors encoded by *ETHYLENE RESPONSE* (*ETR*) genes, and its signal is translated into gene expression changes that regulate other developmental processes. (Left panel) In the absence of ethylene, Constitutive Triple Response 1 (CTR1) is activated by its association with ETR. The active form of CTR1 phosphorylates EIN2, a central positive regulator of the ethylene signalling pathway (Fu *et al*., 2005; Hu *et al*., 2010; Huang *et al*., 2022). The phosphorylation results in EIN2 ubiquitination and subsequent proteolysis by the 26S proteasome (Qiao *et al*., 2009; Ju *et al*., 2012). Similarly, the downstream EIL transcription factors are also targeted for degradation (Guo and Ecker, 2003; Potuschak *et al*., 2003; Gagne *et al*., 2004; Binder *et al*., 2007; An *et al*., 2010). The breakdown of both EIN2 and EILs inhibits signalling and prevents downstream ethylene responses. (Right panel) Upon ethylene binding, the ETR–CTR1 complex is inactive, lowering EIN2 phosphorylation. The unphosphorylated EIN2 then undergoes proteolytic cleavage, which releases the active C-terminal part (EIN2-C) (Qiao *et al*., 2012; Wen *et al*., 2012). Active EIN2-C has two roles in ethylene signalling: (i) forming a complex with EIN2 nuclear-associated protein I (ENAP) to regulate *EIN3/EIL* transcription (Zhang *et al*., 2017) and (ii) forming a complex with mRNAs encoding EBFs and targeting them for degradation (Li *et al*., 2015; Merchante *et al*., 2015). These two roles of EIN2-C complete the ethylene signalling by increasing the expression of *EIN3/EIL* and of ERF (Ethylene Response Factor) transcription factor-encoding genes (Bapat *et al*., 2010). Created in BioRender. Wang, R. (2025) https://BioRender.com/lpjus9o.

Central and of paramount importance for the progression of ripening in tomatoes is the production, sensing, and signal transduction of the gaseous hormone ethylene, as depicted in Fig. 1. As several excellent reviews of these subjects have been published, we focus here on the resulting transcriptional regulation during ripening.

### Ethylene biosynthesis, sensing, and signal transduction

In higher plants, *S*-adenosyl-methionine (SAM) is converted to 1-aminocyclopropane-1-carboxylic acid (ACC), followed by oxidation of ACC by ACC oxidase (ACO) to produce ethylene. The ACC synthase genes *ACS2* and *ACS4* are specifically involved in ripening (Barry *et al*., 2000). The expression of both genes increases sharply during ripening, and their activity at the transcriptional level is co-regulated by the major ripening-regulating TFs discussed later. System 2 ethylene biosynthesis is activated during ripening, concurrent with increased expression of *ACS2* and *ACS4*. In contrast to system 1 biosynthesis, which functions pre-ripening, system 2 is regulated by ethylene through positive feedback during ripening. It is worth noting that both ethylene production and ethylene-forming enzyme activity are different in pericarp and septa on the one hand, and columella and placenta on the other. Ethylene production and ACO activity are higher in the former two tissues, whereas ACS activity and ACC content are higher in the latter tissues, and SAM content is highest in the locular gel. This is possibly explained by more instantaneous turnover of formed ACC by ACO in the pericarp, or by ACC transport from the inner tissues outwards (Van de Poel *et al*., 2014).

The ethylene signalling pathway involves the proteins ETHYLENE RESPONSE (ETR), CONSTITUTIVE TRIPLE RESPONSE1 (CTR1), and ETHYLENE INSENSITIVE2 (EIN2), and is summarized in Fig. 1. In tomato, ETR3 (NR) and ETR4 are the main ethylene receptors that are expressed during ripening (Tieman *et al*., 2000).

Several spontaneous mutations with ripening defects affect ethylene production or signalling. The fruits of the mutants *ripening inhibitor* (*rin*) (Robinson and Tomes, 1968) and *non-ripening* (*nor*) (Tigchelaar *et al*., 1973) do not or only partially ripen and are the best known mutations that are associated with TF activity (R. Wang *et al*., 2020). A third mutation, *Colorless non-ripening* (*Cnr*) (Thompson *et al*., 1999), blocks ripening and is associated with an SPL (SQUAMOSA Promoter-Binding Protein-Like) TF-encoding gene but not with its activity, as CRISPR/Cas-generated knockout mutants show only a very mild, cuticle-related phenotype (Y. Gao *et al*., 2019; Chen *et al*., 2024).

### Regulators of multiple ripening processes

In tomato, some TFs have been reported as major regulators according to the ripening defects in their spontaneous mutants. For instance, the severe non-ripe fruit phenotype of the spontaneous mutants *rin* and *nor* suggest that the TFs MADS-RIN, a SEPALLATA clade (E-class) MADS-domain TF (Vrebalov *et al*., 2002), and NAC (NAM, ATAF1/2, and CUC2)-NOR (Giovannoni *et al*., 2004), whose genes are linked to these mutations, are essential activators of normal ripening. For TFs that do not have naturally occurring mutants, studies have reported a role in fruit ripening through RNA interference (RNAi) knockdown, for example, for *APETALA2a* (*AP2a*) (Karlova *et al*., 2011) and *FUL1/2* (Bemer *et al*., 2012; Shima *et al*., 2013). Later, with the help of gene editing, knockout mutants were obtained to more precisely uncover their function and, for example, allow specific knockouts of very homologous genes (e.g. *FUL1/2*) for the first time (R. Wang *et al*., 2019). The single knockout of *FUL1* or *FUL2* resulted in only mild ripening changes, while the dual knockout of these genes caused more serious defects, so both FUL1 and FUL2 are positive regulators and act redundantly on ripening (R. Wang *et al*., 2019). As its knockdown plants produced more than three times the normal amount of ethylene during ripening, AP2a was described as a repressor of ethylene biosynthesis and, thus, ripening (Chung *et al*., 2010; Karlova *et al*., 2011). An additional advantage of using knockout mutations over RNAi or virus-induced gene silencing (VIGS) is that it can reveal feedback self-regulation of expression, as was the case for the negative feedback of functional AP2a on its own gene expression (R. Wang *et al*., 2019). AP2a was proven to promote brassinosteroid (BR) synthesis via AP2a-dependent transcriptional activation of *DWARF* (*DWF*) expression (Sang *et al*., 2022). Additional regulators, such as TAGL1 and the HD-Zip homeobox protein LeHB-1, probably function in specifying organ identity, although they are also regulated during ripening (Lin *et al*., 2008; Itkin *et al*., 2009; Vrebalov *et al*., 2009). Whereas the crucial role of RIN is undisputed, MADS-domain proteins usually act as homodimers, heterodimers, or multimers. In yeast-two-hybrid experiments, RIN associated with itself as well as with FUL1, FUL2, MBP3, TAG1, and TAGL1 (Leseberg *et al*., 2008; Jiang *et al*., 2022). EIN3-like (EIL) TFs directly regulate *NOR*, *RIN*, and *FUL1* expression through binding to their promoters (demonstrated for EIL1), and this appears to favour FUL1 as the partner (Huang *et al*., 2022). RIN and TAGL1 in combination regulated *ACS2* and *ACO1* expression and ethylene production in *in vitro* dropout experiments, which shows that TAGL1 can be a functional partner in an EIN3–MADS loop (Lü *et al*., 2018).

### Ethylene response factors and auxin response factors

As their name implies, ethylene response factors (ERFs) are TFs that have been implicated in the transcriptional response to ethylene, although the proteins of this group in tomato are classified as such because of their conserved ERF domain, rather than because their response to ethylene has always been established. Analysis of the tomato genome identified 77 *ERF* genes in nine subclasses (A–J) (Liu *et al*., 2016) and, in a later analysis, as many as 143 genes (Zhang *et al*., 2022). Apart from their role in fruit ripening, earlier work has identified functions of ERFs in disease resistance and stress responses. As is often the case, the first hints of a role in ripening come from their elevated expression during ripening, which is absent in the general ripening mutants *rin*, *nor*, and *Nr* (*Never-ripe*). Others are down-regulated during ripening and may have a negative, modulating effect on ripening. Among four genes identified as being up-regulated during ripening, *ERF.E1*, *E2*, *E4*, and *F2*, the first three are up-regulated by external ethylene and directly regulated by MADS-RIN. Down-regulation of *ERF.E4* (*LeERF6*) by RNAi increased ethylene and carotenoid production by up-regulating *ACS2* and several carotenoid pathway genes (Lee *et al*., 2012).

*SlERF.D7* is up-regulated relatively late in ripening, and RNAi reduced ethylene production and partially inhibited ripening (Gambhir *et al*., 2022). Antisense suppression of ERF.H1 (LeERF1) slightly decreased ethylene production but, curiously, resulted in the up-regulation of several downstream ripening-related genes (Li *et al*., 2007).

Eleven ERF proteins have one or two typical EAR (Ethylene-responsive element binding factor-associated Amphiphilic Repression; LxLxL or DLNxxP) motifs and are likely repressors of transcription through histone modification (see Box 1). One example is ERF.F12, whose gene expression decreases during the transition to ripening, and its knockout results in earlier fruit ripening (Deng *et al*., 2022).

SlERF.G3-Like governs a hierarchical transcriptional cascade regulating tomato fruit ripening by directly manipulating ethylene production and the phenylpropanoid pathway, while MADS-RIN directly activates it simultaneously (You *et al*., 2024). Similarly, the knockout of *SlERF.D6*, which positively influences ripening initiation and carotenoid accumulation through a cascade consisting of the TF SlDEAR2 and the TCP (Teosinte branched1/Cycloidea/Proliferating cell factor) TF SlTCP12, delays the onset of ripening, the formation of carotenoids, and softening, and also delays and attenuates ethylene production (Chen *et al*., 2025).

ARFs are not only involved in fruit set and growth but also play roles in ripening regulation, providing crosstalk between auxin and ethylene signalling. The ARF2 orthologs ARF2A and 2B are differently positively regulated by exogenous ethylene (ARF2A) and auxin (ARF2B), and their combined knockdown by RNAi resulted in lower ethylene and carotenoid production and enhanced firmness. The latter phenotypes could not be complemented with exogenous ethylene, prompting the suggestion that besides the reduction in ethylene production, ethylene sensitivity was reduced (Hao *et al*., 2015). The down-regulation by antisense of *ARF4/DR12*, whose expression is up-regulated during ripening, resulted in dark green immature fruits, which became blotchy during ripening (Jones *et al*., 2002).

### NAC transcription factors

Besides the NAC-NOR TF described above as a major regulator of ripening, in the past years, several additional NAC TFs with a role in ripening have been described (Forlani *et al*., 2021). Foremost of these, the closest homolog of *NOR* in tomato is *NOR-LIKE1* (*NL1*) (Gao *et al*., 2018), previously also described as *SlNAC3*. VIGS down-regulation or knockout mutation of *NL1* resulted in delayed onset of ripening, reduced ethylene production, reduced *ACS2/4* expression, and reduced carotenoid synthesis (Gao *et al*., 2018). Given the close similarity of *NL1* to *NOR*, it is not surprising that the two genes may have overlapping functions, although *NL1* is expressed throughout fruit development, whereas *NOR* expression occurs almost exclusively during ripening. Other members of the tomato NAC family, such as NAM1 (Gao *et al*., 2021) and NAC4 (Zhu *et al*., 2014), also have positive ripening regulatory properties, albeit weaker than those of NL1 or NOR.

### Towards a comprehensive, hierarchical model of the regulation of ripening

**Fig. 2.**
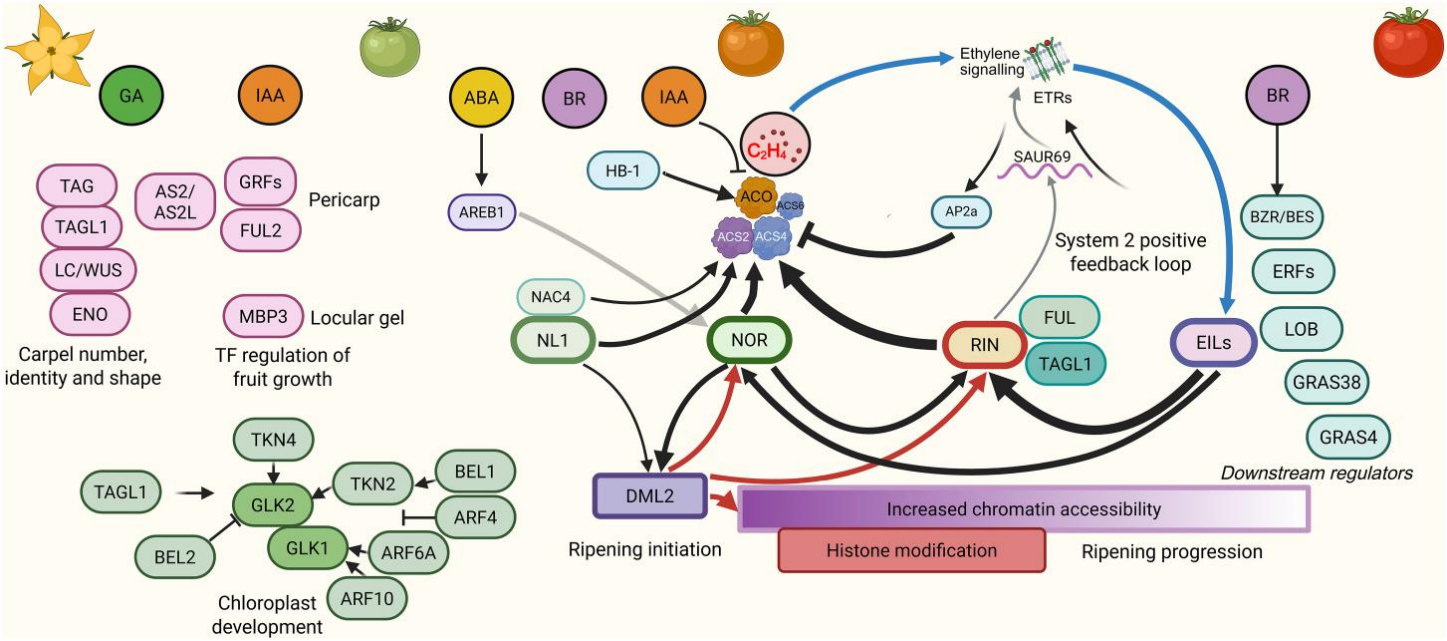
Transcriptional regulation network of fruit development, ripening initiation, and ripening progression. Transcription factors involved in fruit development and ripening are depicted as rounded rectangles in a putative temporal or developmental sequence from left to right. Arrows emanating from these TFs indicate transcriptional regulation. The thickness of the arrows shows the predicted strength of regulation. Arrows from C_2_H_4_ to EILs represent ethylene signal transduction. Arrows emanating from DML2 denote DNA cytosine demethylation. Circles depict phytohormones: ABA, abscisic acid; BR, brassinosteroids; GA, gibberellic acid; IAA, auxin. Created in BioRender. Wang, R. (2025) https://BioRender.com/4qtac55.

Recently, the integration of data from mutants of the major regulatory genes *NL1*, *NAC-NOR*, and *MADS-RIN*, along with knowledge of their effects on downstream gene expression, ethylene production, and fruit colour and firmness, has led to a more comprehensive model of the transcriptional regulation of ripening (Aprilyanto *et al*., 2025). Thus, ripening is initiated mainly by the action of NL1, which is expressed throughout fruit development, and subsequently, after its induction, by NAC-NOR, through the activation of *ACS2*, resulting in ethylene production. Since *nl1* mutants show merely a delay in, not a block of, ripening initiation, which can be complemented by exogenous ethylene, other minor TFs such as NAC4 may be responsible for the residual initiating activity. NAC-NOR activity is required to sustain elevated ethylene during ripening, which in turn induces MADS-RIN expression and a boost in ethylene production through *ACS4*, which is apparently required for full ripening progression. All four single mutants are capable of initiating ripening and demonstrate relative contributions to ethylene production, from low but essential for NL1 and possibly NAC4, to intermediate for NAC-NOR, and large for MADS-RIN. A double mutant of the putative paralogs *NL1* and *NAC-NOR* does not initiate ripening at all up to 340 days post anthesis, and resembles the original spontaneous *nor* mutant (*nor-s*), which we now know has dominant negative-like TF activity because it lacks a transcriptional activation domain. This suggests, for the wild type, a required regulatory interaction between NL1 and NAC-NOR activity for normal ripening initiation, which is blocked by the nor-s protein once it starts to form at the initiation of ripening. To add to the complexity, some or all of these TFs also respond to ethylene in a positive feedback loop (Fig. 2).

An additional remaining question relates to the down-regulation of ethylene production after it has peaked. Possibly, there is a role for the negative regulatory TF AP2a, which itself is induced by ethylene during ripening and has a negative-feedback effect on ethylene production (Chung *et al*., 2010). Curiously, the expression of the ethylene receptor genes *ETR3* and *ETR4* follows the climacteric ethylene peak. Since these receptors inhibit the ethylene response, their up-regulation would reduce sensitivity to ethylene and might initiate the down-regulation of ethylene production (Mata *et al*., 2018).

### Other hormones and their interaction with regulators of development and ripening

Apart from the essential roles of gibberellin, cytokinin, and auxin in fruit set and growth, all three have a negative effect on ethylene production, and hence, they delay ripening. The auxin-mediated negative effect on ethylene production appears to be negated by increased ethylene sensitivity through the induction of *SMALL AUXIN-UP RNA 69* (*SAUR69*), a target of *rin-mc* (the spontaneous mutant), at the onset of ripening (Shin *et al*., 2019). Abscisic acid production peaks prior to the initiation of ripening and promotes it, likely through the binding of ABA-RESPONSIVE ELEMENT BINDING PROTEIN 1 (AREB1) to the promoter of *NAC-NOR* (Mou *et al*., 2018). BRs, through the BRZ1/BES1 TF family, play a positive role in carotenoid production and softening (see later).

### The fringes of the main regulatory network

Although several uses of the term ‘effector’ exist in biology, here, in the context of ripening regulation, we define developmental effector genes as those genes at the end of a transcriptional regulatory cascade or network, which themselves do not encode a TF or TF-interacting factor. This places them at the end of, but does not include them in, so-called gene regulatory networks (GRNs) (L. Wang *et al*., 2019), as discussed below. The major regulators of tomato fruit ripening regulate the expression of downstream effector genes, either directly through binding to the promoter to activate or inhibit a gene’s transcription, or indirectly, through the activation or inhibition of ethylene production with its own downstream regulatory circuit, or through regulating the expression of secondary TFs that in turn regulate effector genes. Effector genes function, for example, through their product’s enzymatic activity, to regulate the processes that are known to be characteristics of ripe fruit, namely colour development, softening, and flavour development. Mostly one-to-one regulatory relationships have been explored so far; however, instead of hierarchical linear regulatory interactions, TFs work in networks to control the expression of effector genes and each other—the so-called GRNs (Karlova *et al*., 2014).

On the one hand, multiple TFs have targets in common, sometimes redundantly, which may act to buffer against disruption by single mutations. For example, the key genes in ethylene biosynthesis, *ACS2* and *ACS4*, are direct targets of MADS-RIN (Fujisawa *et al*., 2011 , 2013), NOR, and NL1 (Gao *et al*., 2018). TFs sometimes form a complex to regulate a gene. MADS-domain TFs usually form dimers and tetramers to bind to the promoters (Riechmann *et al*., 1996; Theißen and Saedler, 2001). Thus, TAGL1, MADS-RIN, FUL1, and FUL2 probably regulate downstream gene transcription by forming dimers or tetramers, possibly in varying compositions (Fujisawa *et al*., 2014). On the other hand, one TF can regulate multiple genes in different pathways. In addition to ethylene biosynthesis, MADS-RIN directly regulates *PSY1* in lycopene biosynthesis and *CEL2* in cell wall degradation (Fujisawa *et al*., 2011 , 2013); the regulation of these genes was confirmed by the sharp decrease in their expression in knockout mutants of *MADS-RIN* (Ito *et al*., 2017). NAC TFs can also dimerize, and may require such interaction to activate transcription. An interaction between the truncated NOR product of the spontaneous *nor* allele, resulting in an inhibiting dimer, may underlie the *trans*-dominant negative action of *nor* (Y. Gao *et al*., 2019; R. Wang *et al*., 2019). However, the dimer formation of tomato NAC TFs and their possible effect on transcriptional activation have not been explored until now.

In tomato, additional regulators include the GRAS family TFs GRAS4 (Y. Liu *et al*., 2021) and GRAS38/SlFSR (Fruit Shelf-life Regulator) (Shinozaki *et al*., 2018; L. Zhang *et al*., 2018; Shen *et al*., 2024), the ARF family TFs ARF4 (Jones *et al*., 2002) and ARF7 (de Jong *et al*., 2011), and the HD-Zip homeobox proteins LeHB-1 (Lin *et al*., 2008) and VAHOX1 (F. Li *et al*., 2023).

## Downstream events, colour, metabolism, flavour, and firmness

The major regulators of tomato fruit ripening directly or indirectly regulate the expression of downstream effector genes, as described in the previous section. Effector genes may be directly involved, for example, through their product’s enzymatic activity, in the processes of colour development, softening, and flavour development that take place during ripening (Karlova *et al*., 2014). The recent study of various combinations of knockout alleles of *NL1*, *NAC*-*NOR*, and *MADS*-*RIN* suggests that colour development and a decrease in fruit firmness closely follow ethylene production, and only an (almost) complete abrogation of climacteric ethylene production in *nor/nl1* and *nor/rin* double mutants blocks these processes (Aprilyanto *et al*., 2025).

### Colour

During ripening, with the accumulation of carotenoids and the degradation of chlorophyll (Cunningham and Gantt, 1998), when chloroplasts turn into chromoplasts, the fruit colour changes from green to red, via yellow and orange. The formation, size, and number of chloroplasts developing prior to the onset of ripening are discussed later (see Metabolism and flavour); the import of nuclear genome-encoded carotene biosynthesis pathway proteins is required and, probably through carotenoid biosynthesis, drives the conversion of chloroplasts into chromoplasts. Although numerous TFs are known to regulate chloroplast formation and proliferation, thereby indirectly impacting on carotenoid accumulation during ripening, no TFs that directly control the conversion to chromoplasts are currently known.

The red colour in a ripe tomato derives from lycopene, which accounts for more than 90% of the carotenoids (Ronen *et al*., 2000). BRs interact with ethylene to regulate fruit ripening. The TF BRASSINAZOLE-RESISTANT 1 (BZR1) can contribute to the ethylene burst and carotenoid accumulation by directly targeting ethylene and carotenoid biosynthetic genes, and the exogenous BRs promote fruit ripening in a dose-dependent manner (He *et al*., 2021; Meng *et al*., 2023). AP2 is a TF family involved in tomato fruit ripening with five homologs (Chung *et al*., 2020). The EAR motif of AP2 can recruit the co-repressor TPL, forming a complex with histone deacetylase to reduce the histone acetylation of downstream effector genes. AP2a has been proven to play a positive role in fruit ripening, but SlAP2c is a repressor, and they orchestrate a transcriptional cascade in tomato carotenoid biosynthesis (He *et al*., 2024). The TF WRKY32 regulates tomato fruit colour by up-regulating *YELLOW FRUITED-TOMATO 1* (*YFT1*), encoding the EIN2 protein, a key protein in ethylene signalling (Zhao *et al*., 2021).

Cultivated tomatoes do not synthesize anthocyanin as the pathway ends at naringenin, which is yellow, but the introgression of the dominant mutation *Anthocyanin fruit* (*Aft*) from *Solanum chilense* induces anthocyanin accumulation in the tomato fruit peel (Jones, 2003).

### Texture and firmness

Fruit firmness is an important trait in tomato production, as firmer texture reduces losses during long-distance transport and extends shelf life. Fruit texture changes from firm at the beginning of ripening (breaker stage) to soft when the fruits are completely ripe. Cell wall modification, resulting from structural changes in polysaccharides (Hyodo *et al*., 2013) that include the degradation of pectin, cellulose and hemicelluloses, is the main cause of change in fruit texture (Peng *et al*., 2022).

More than 50 cell wall structure-related genes have been annotated in the tomato genome (Wang and Seymour, 2022), including those encoding enzymes that digest polysaccharides, which function as effector genes targeted by TFs, such as *PECTATE LYASE* (*PL*) (Uluisik *et al*., 2016) and *CELLULASE 2* (*CEL2*) (Lashbrook *et al*., 1994). Expression of these genes goes up dramatically (more than 5000-fold) during ripening (X. Wang *et al*., 2020), consistent with the change in fruit texture from firm to soft. Besides enzyme-encoding genes, there are some TFs that have been reported to be involved in the regulation of cell wall modification. BRI1-EMS-SUPPRESSOR1 (BES1) and LATERAL ORGAN BOUNDARIES (LOB1) control the cell wall firmness of tomato fruits by inhibiting the transcription of the pectin methylesterase-encoding gene *PMEU1*, whose activity confers cross-linking ability on the pectin network of the cell wall, and the wall-loosening protein EXPANSIN 1-encoding gene *EXP1* (Shi *et al*., 2021; H. Liu *et al*., 2021). Besides *EXP1* and endoglucanase *CEL2*, the downstream effector genes of LOB1 regulate cell wall disassembly synergistically; knocking out both of these genes enhances fruit firmness without affecting other aspects of fruit quality (Su *et al*., 2023).

### Metabolism and flavour

*3-Dehydroquinate dehydratase/shikimate dehydrogenase* (*DQD/SDH*) encodes a rate-limiting enzyme catalyzing shikimate synthesis, and the TF TAGL1 controls flavonoid biosynthesis during ripening by regulating *SlDQD/SDH2* (Wang *et al*., 2023). The TF SlMYB12 is involved in tomato naringenin chalcone biosynthesis (Ballester *et al*., 2009). SPL-CNR could repress *MYB12* transcription to negatively regulate flavonoid biosynthesis (Zhou *et al*., 2024). MYC2 is a basic helix-loop-helix (bHLH) TF that forms a complex with mediator MED25 to activate the transcription of *SlMYB12*, which plays a crucial role in the accumulation of flavonoids and orchestrates a hierarchical transcriptional cascade regulating fruit flavonoid metabolism in tomato (Deng *et al*., 2024).

Chloroplasts are present in immature fruits and actively photosynthesize during fruit development. Since these are the organelles that produce sugars and accumulate starch, which during ripening is converted to sugars, the regulation of chloroplast proliferation impacts on the final fruit sugar and carotenoid content (see Colour, earlier). *TAGL1*, possibly through its role as an organ identity gene, also plays a role in fruit chloroplast density. Knockout mutants of *TAGL1* are darker green, and the epigenetic mutation *GREEN STRIPE* (*GS*), which leads to dark green stripes in a lighter wild-type background of developing fruits, involves hypermethylation of the *TAGL1* promoter and its lower expression (Liu *et al*., 2020). The TF GOLDEN2-LIKE 2/UNIFORM (GLK2) is expressed in a latitudinal gradient, decreasing from the crown end to the blossom end of the fruit; in this gradient, decreased expression leads to lower chloroplast density and the GREEN SHOULDER phenotype of wild-type tomato (Powell *et al*., 2012; Nguyen *et al*., 2014). The *GLK2* expression gradient is controlled by *KNOTTED1-LIKE HOMEOBOX (KNOX)-*class genes *TKN4/UNIFORM GRAY GREEN* and *TKN2* (Nadakuduti *et al*., 2014). In turn, *GLK2* expression is repressed by BEL1-LIKE HOMEODOMAIN 2 (BEL2), which decreases the green shoulder formation, through *GLK2* promoter binding and direct interaction of BEL2 with the GLK2 protein (Niu *et al*., 2022). Another BEL protein, BEL11, regulates chloroplast development through binding to the *TKN2* promoter (Meng *et al*., 2018). Chloroplast development is linked to auxin signalling through the activity of ARF4, ARF6A, and ARF10. ARF4/DEVELOPMENTALLY REGULATED 12 (DR12) acts as a repressor of auxin signalling, and its knockout results in darker green fruits and the up-regulation of *AGPase*, a major gene in starch accumulation (Sagar *et al*., 2013). ARF6A and ARF10 are positive regulators of chloroplast development and, hence, sugar accumulation, possibly through the regulation of *GLK1* expression (Yuan *et al*., 2018, 2019) (Fig. 2).

A sweet tomato is normally more welcomed by consumers than one that is not sweet, so breeding sweeter tomatoes is always an important goal for breeders. Sugar transporters and invertases are key players in sugar accumulation in tomato fruits. Sugars Will Eventually be Exported Transporters (SWEETs) have a vital function in sugar transport by facilitating the movement of sugars across cell membranes, and silencing *SlSWEET7a* or *SlSWEET14* enhanced sugar content in tomato fruits (Jeena *et al*., 2019; Zhang *et al*., 2021). *SWEETs* are regulated by TFs that bind to their *cis*-element; for example, ELONGATED HYPOCOTYL5 (HY5) binds directly to the G-box of *SWEET12c*, repressing the accumulation of sugar by activating *SWEET12c* expression, so the *HY5* knockout mutant in ‘Ailsa Craig’ tomato could increase the fructose and glucose contents of fruits by ∼20% (Jia *et al*., 2024). Meanwhile, SlMYB1R1, a MYB TF that is highly expressed during fruit development, also regulates sugar accumulation in tomato fruits by modulating *SWEET12c* expression (Liu *et al*., 2025).

## Conclusions and remaining questions

Fruit development and ripening regulation at the molecular level has been studied for a long time, and several major and many other regulatory genes have been identified by classical forward and reverse genetics methods. This identification and discovery has lately been accelerated by CRISPR/Cas mutagenesis. Besides simply knocking out genes, it is possible to edit *cis*-regulatory elements (CREs) in promoters to fine-tune gene expression (Tang and Zhang, 2023) or knock-in enhancers (Yao *et al*., 2024). With an increasing number of gene functions being studied in detail and higher efficiency of gene editing, the possibility of designing a gene and a tomato with a specific colour (Yang *et al*., 2023) or flavour becomes practical in the future. Some spontaneous ripening mutations have been studied extensively, although their nature has been elucidated only relatively recently. The interactions of spontaneous alleles and the role of multiple TFs, CREs in gene promoters, and microRNAs in ripening have been evaluated in this review, taking us a step further in understanding the entire regulatory network. However, the regulation of development and ripening, which consists of many factors and different layers of regulation at the genetic and epigenetic, transcriptional and post-transcriptional, translational and post-translational levels, and *in trans* or *in cis*, is still very complex. Some outstanding questions, not exhaustively listed here, are the relative importance, regulatory hierarchy, and timing of the main TFs affecting climacteric ethylene production, which have only recently become clearer (Fig. 2). Since ethylene production peaks instead of levelling off, how its production is shut down remains unclear. Furthermore, as we know now, most components reciprocally affect each other’s expression, possibly through varying protein–protein interactions that are still largely unexplored, and so the number of regulatory interactions can become practically infinite. Furthermore, as the regulatory network of fruit ripening regulation is emerging, attention may shift to the fruit growth regulatory network. Although many TFs involved in fruit size have been identified (Fig. 2), it is unclear how these, at the gene expression level, affect the activity of cell cycle genes that are the basis of cell proliferation and expansion.

While, for example, the regulation of multiple genes and GRNs at the transcript level can be inferred by approaches such as co-expression analysis or weighted gene co-expression network analysis (WGCNA), the next challenge that emerges is the integration, in models, of large amounts of data from multi-omics approaches in multiple dimensions, such as high-throughput genomics, transcriptomics, proteomics, metabolomics, and epigenomics. The analysis of these data requires effective strategies. Possibly, the use of artificial intelligence (AI) and machine learning (ML) will help to map these interactions and predict the effects of perturbations in them. For example, by learning from experimental protein structures deposited in databases and being trained to high accuracy, AlphaFold leveraged multi-sequence alignments into a deep learning algorithm and released over 200 million predictions of protein structures (Jumper *et al*., 2021). Most AI and ML applications in biology have, so far, been developed for human cells and diseases, such as a platform predicting Alzheimer’s disease markers with AI-assisted multi-omics analysis, and a model uncovering transcriptional regulations across 213 human cell types (Fu *et al*., 2025; Latifi-Navid *et al*., 2025). Nowadays, AI is revolutionizing precision crop breeding by characterizing genomic big data of germplasm resources, digitalizing the collection of phenotyping data, generating models to explain genomic data, and integrating multi-omics big data (Farooq *et al*., 2024). Thus, AI and ML could also help to build a comprehensive regulatory network model, which could not only help reveal the complex interactions between TFs and their upstream regulation and downstream effects but also guide practical applications in tomato breeding. The ability to predict the effects of perturbations in this complex system may help in the rational choice of combinations of mutant alleles or natural variants with traits that are not obviously related. Moreover, it has become clear that many variants and genes underlying beneficial quantitative trait loci are not mere knockouts of protein function, but rather much more often have a quantitative effect on gene expression (Swinnen *et al*., 2016). Considering that many of the genes discussed here, particularly those regulating ripening downstream of these regulators (Fig. 2), are subject to regulation by multiple TFs, one could start to predict which modifications in binding sites in a gene promoter, or combinations thereof, would result in the desired quantitative response and trait. We predict that even if no such platforms or models of tomato fruit development and ripening regulation are available now, they will appear soon.

## Data Availability

No new data was generated for this manuscript.

## References

[eraf357-B1] Aflitos S, Schijlen E, de Jong H, et al 2014. Exploring genetic variation in the tomato (*Solanum* section *Lycopersicon*) clade by whole-genome sequencing. The Plant Journal 80, 136–148. 25039268 10.1111/tpj.12616

[eraf357-B2] Alexander L, Grierson D. 2002. Ethylene biosynthesis and action in tomato: a model for climacteric fruit ripening. Journal of Experimental Botany 53, 2039–2055. 12324528 10.1093/jxb/erf072

[eraf357-B3] Alonge M, Wang X, Benoit M, et al 2020. Major impacts of widespread structural variation on gene expression and crop improvement in tomato. Cell 182, 145–161. 32553272 10.1016/j.cell.2020.05.021 PMC7354227

[eraf357-B4] An F, Zhao Q, Ji Y, et al 2010. Ethylene-induced stabilization of ETHYLENE INSENSITIVE3 and EIN3-LIKE1 is mediated by proteasomal degradation of EIN3 binding F-box 1 and 2 that requires EIN2 in *Arabidopsis*. The Plant Cell 22, 2384–2401. 20647342 10.1105/tpc.110.076588 PMC2929093

[eraf357-B5] Aprilyanto V, Wang X, Wang R, Kronenberg S, Bos F, Peña-Ponton C, Angenent GC, de Maagd RA. 2025. A comprehensive model of tomato fruit ripening regulation by the transcription factors NOR-like1, NAC-NOR, and MADS-RIN. Plant Physiology 198, kiaf291. 40587418 10.1093/plphys/kiaf291 PMC12305540

[eraf357-B6] Ballester A, Molthoff J, de Vos R, et al 2009. Biochemical and molecular analysis of pink tomatoes: deregulated expression of the gene encoding transcription factor SlMYB12 leads to pink tomato fruit color. Plant Physiology 152, 71–84. 19906891 10.1104/pp.109.147322 PMC2799347

[eraf357-B7] Bapat VA, Trivedi PK, Ghosh A, Sane VA, Ganapathi TR, Nath P. 2010. Ripening of fleshy fruit: molecular insight and the role of ethylene. Biotechnology Advances 28, 94–107. 19850118 10.1016/j.biotechadv.2009.10.002

[eraf357-B8] Barry CS, Llop-Tous MI, Grierson D. 2000. The regulation of 1-aminocyclopropane-1-carboxylic acid synthase gene expression during the transition from system-1 to system-2 ethylene synthesis in tomato. Plant Physiology 123, 979–986. 10889246 10.1104/pp.123.3.979 PMC59060

[eraf357-B9] Bemer M, Karlova R, Ballester AR, Tikunov YM, Bovy AG, Wolters-Arts M, Rossetto PDB, Angenent GC, de Maagd RA. 2012. The tomato FRUITFULL homologs TDR4/FUL1 and MBP7/FUL2 regulate ethylene-independent aspects of fruit ripening. The Plant Cell 24, 4437–4451. 23136376 10.1105/tpc.112.103283 PMC3531844

[eraf357-B10] Bergervoet JHW, Berhoeven HA, Gilissen LJW, Bino RJ. 1996. High amounts of nuclear DNA in tomato (*Lycopersicon esculentum* Mill.) pericarp. Plant Science 116, 141–145.

[eraf357-B11] Binder BM, Walker JM, Gagne JM, Emborg TJ, Hemmann G, Bleecker AB, Vierstra RD. 2007. The *Arabidopsis* EIN3 binding F-box proteins EBF1 and EBF2 have distinct but overlapping roles in ethylene signaling. The Plant Cell 19, 509–523. 17307926 10.1105/tpc.106.048140 PMC1867343

[eraf357-B12] Birney E, Stamatoyannopoulos JA, Dutta A, et al 2007. Identification and analysis of functional elements in 1% of the human genome by the ENCODE pilot project. Nature 447, 799–816. 17571346 10.1038/nature05874 PMC2212820

[eraf357-B13] Carvalho A, Vicente MH, Ferigolo LF, et al 2025. The miR319-based repression of SlTCP2/LANCEOLATE activity is required for regulating tomato fruit shape. The Plant Journal 121, e17174. 39590512 10.1111/tpj.17174

[eraf357-B14] Chakrabarti M, Zhang N, Sauvage C, et al 2013. A cytochrome P450 regulates a domestication trait in cultivated tomato. Proceedings of the National Academy of Sciences, USA 110, 17125–17130. 10.1073/pnas.1307313110 PMC3801035 24082112

[eraf357-B15] Chen D, Wang T, Huang H, et al 2024. SlCNR regulates postharvest water loss and wax accumulation in tomato fruit and directly represses the transcription of very-long-chain (VLC) alkane biosynthesis-related genes *SlCER1-2* and *SlCER6*. Postharvest Biology and Technology 208, 112641.

[eraf357-B16] Chen Y, Wang X, Colantonio V, et al 2025. Ethylene response factor SlERF.D6 promotes ripening in part through transcription factors SlDEAR2 and SlTCP12. Proceedings of the National Academy of Sciences, USA 122, 2017. 10.1073/pnas.2405894122 PMC11848416 39928866

[eraf357-B17] Chirinos X, Ying S, Rodrigues MA, et al 2023. Transition to ripening in tomato requires hormone-controlled genetic reprogramming initiated in gel tissue. Plant Physiology 191, 610–625. 36200876 10.1093/plphys/kiac464 PMC9806557

[eraf357-B18] Chung M-Y, Vrebalov J, Alba R, Lee J, McQuinn R, Chung J-D, Klein P, Giovannoni J. 2010. A tomato (*Solanum lycopersicum*) *APETALA2/ERF* gene, *SlAP2a*, is a negative regulator of fruit ripening. The Plant Journal 64, 936–947. 21143675 10.1111/j.1365-313X.2010.04384.x

[eraf357-B19] Chung M-Y, Nath UK, Vrebalov J, Gapper N, Lee JM, Lee D-J, Kim CK, Giovannoni J. 2020. Ectopic expression of miRNA172 in tomato (*Solanum lycopersicum*) reveals novel function in fruit development through regulation of an *AP2* transcription factor. BMC Plant Biology 20, 283. 32560687 10.1186/s12870-020-02489-y PMC7304166

[eraf357-B20] Cong B, Barrero LS, Tanksley SD. 2008. Regulatory change in YABBY-like transcription factor led to evolution of extreme fruit size during tomato domestication. Nature Genetics 40, 800–804. 18469814 10.1038/ng.144

[eraf357-B21] Cunningham FX, Gantt E. 1998. Genes and enzymes of carotenoid biosynthesis in plants. Annual Review of Plant Physiology and Plant Molecular Biology 49, 557–583. 10.1146/annurev.arplant.49.1.557 15012246

[eraf357-B22] de Jong M, Wolters-Arts M, García-Martínez JL, Mariani C, Vriezen WH. 2011. The *Solanum lycopersicum* AUXIN RESPONSE FACTOR 7 (SlARF7) mediates cross-talk between auxin and gibberellin signalling during tomato fruit set and development. Journal of Experimental Botany 62, 617–626. 20937732 10.1093/jxb/erq293 PMC3003806

[eraf357-B23] Deng H, Chen Y, Liu Z, et al 2022. SlERF.F12 modulates the transition to ripening in tomato fruit by recruiting the co-repressor TOPLESS and histone deacetylases to repress key ripening genes. The Plant Cell 34, 1250–1272. 35099538 10.1093/plcell/koac025 PMC8972228

[eraf357-B24] Deng H, Wu M, Wu Y, Xiao X, Gao Z, Li H, Hu N, Gao Y, Grierson D, Liu M. 2024. SlMYC2-SlMYB12 module orchestrates a hierarchical transcriptional cascade that regulates fruit flavonoid metabolism in tomato. Plant Biotechnology Journal 23, 477. 39506604 10.1111/pbi.14510 PMC11772319

[eraf357-B25] Domínguez M, Dugas E, Benchouaia M, Leduque B, Jiménez-Gómez JM, Colot V, Quadrana L. 2020. The impact of transposable elements on tomato diversity. Nature Communications 11, 4058. 10.1038/s41467-020-17874-2 PMC7426864 32792480

[eraf357-B26] Dong R, Yuan Y, Liu Z, Sun S, Wang H, Ren H, Cui X, Li R. 2023. *ASYMMETRIC LEAVES 2* and *ASYMMETRIC LEAVES 2*-LIKE are partially redundant genes and essential for fruit development in tomato. The Plant Journal 114, 1285–1300. 36932869 10.1111/tpj.16193

[eraf357-B27] Farooq MA, Gao S, Hassan MA, Huang Z, Rasheed A, Hearne S, Prasanna B, Li X, Li H. 2024. Artificial intelligence in plant breeding. Trends in Genetics 40, 891–908. 39117482 10.1016/j.tig.2024.07.001

[eraf357-B28] Fernández-Lozano A, Yuste-Lisbona FJ, Pérez-Martín F, Pineda B, Moreno V, Lozano R, Angosto T. 2015. Mutation at the tomato *EXCESSIVE NUMBER OF FLORAL ORGANS* (*ENO*) locus impairs floral meristem development, thus promoting an increased number of floral organs and fruit size. Plant Science 232, 41–48. 25617322 10.1016/j.plantsci.2014.12.007

[eraf357-B29] Ferreira e Silva GF, Silva EM, Azevedo MdS, Guivin MA, Ramiro DA, Figueiredo CR, Carrer H, Peres LE, Nogueira FT. 2014. microRNA156-targeted SPL/SBP box transcription factors regulate tomato ovary and fruit development. The Plant Journal 78, 604–618. 24580734 10.1111/tpj.12493

[eraf357-B30] Forlani S, Mizzotti C, Masiero S. 2021. The NAC side of the fruit: tuning of fruit development and maturation. BMC Plant Biology 21, 238. 34044765 10.1186/s12870-021-03029-y PMC8157701

[eraf357-B31] Fu D, Zhu B, Zhu H, Jiang W, Luo Y. 2005. Virus-induced gene silencing in tomato fruit. The Plant Journal 43, 299–308. 15998315 10.1111/j.1365-313X.2005.02441.x

[eraf357-B32] Fu X, Mo S, Buendia A, et al 2025. A foundation model of transcription across human cell types. Nature 637, 965–973. 39779852 10.1038/s41586-024-08391-z PMC11754112

[eraf357-B33] Fujisawa M, Nakano T, Ito Y. 2011. Identification of potential target genes for the tomato fruit-ripening regulator RIN by chromatin immunoprecipitation. BMC Plant Biology 11, 26. 21276270 10.1186/1471-2229-11-26 PMC3039564

[eraf357-B34] Fujisawa M, Nakano T, Shima Y, Ito Y. 2013. A large-scale identification of direct targets of the tomato MADS box transcription factor RIPENING INHIBITOR reveals the regulation of fruit ripening. The Plant Cell 25, 371–386. 23386264 10.1105/tpc.112.108118 PMC3608766

[eraf357-B35] Fujisawa M, Shima Y, Nakagawa H, Kitagawa M, Kimbara J, Nakano T, Kasumi T, Ito Y. 2014. Transcriptional regulation of fruit ripening by tomato FRUITFULL homologs and associated MADS box proteins. The Plant Cell 26, 89–101. 24415769 10.1105/tpc.113.119453 PMC3963596

[eraf357-B36] Gagne JM, Smalle J, Gingerich DJ, Walker JM, Yoo S, Yanagisawa S, Vierstra RD. 2004. *Arabidopsis* EIN3-binding F-box 1 and 2 form ubiquitin-protein ligases that repress ethylene action and promote growth by directing EIN3 degradation. Proceedings of the National Academy of Sciences, USA 101, 6803–6808. 10.1073/pnas.0401698101 PMC404126 15090654

[eraf357-B37] Gambhir P, Singh V, Parida A, Raghuvanshi U, Kumar R, Sharma AK. 2022. Ethylene response factor ERF.D7 activates *auxin response factor 2* paralogs to regulate tomato fruit ripening. Plant Physiology 190, 2775–2796. 36130295 10.1093/plphys/kiac441 PMC9706452

[eraf357-B38] Gao C, Ju Z, Cao D, Zhai B, Qin G, Zhu H, Fu D, Luo Y, Zhu B. 2015. MicroRNA profiling analysis throughout tomato fruit development and ripening reveals potential regulatory role of RIN on microRNAs accumulation. Plant Biotechnology Journal 13, 370–382. 25516062 10.1111/pbi.12297

[eraf357-B39] Gao L, Gonda I, Sun H, et al 2019. The tomato pan-genome uncovers new genes and a rare allele regulating fruit flavor. Nature Genetics 51, 1044–1051. 31086351 10.1038/s41588-019-0410-2

[eraf357-B40] Gao Y, Wei W, Zhao X, et al 2018. A NAC transcription factor, NOR-like1, is a new positive regulator of tomato fruit ripening. Horticulture Research 5, 75. 30588320 10.1038/s41438-018-0111-5 PMC6303401

[eraf357-B41] Gao Y, Zhu N, Zhu X, et al 2019. Diversity and redundancy of the ripening regulatory networks revealed by the fruitENCODE and the new CRISPR/cas9 *CNR* and *NOR* mutants. Horticulture Research 6, 39. 30774962 10.1038/s41438-019-0122-x PMC6370854

[eraf357-B42] Gao Y, Fan Z, Zhang Q, et al., 2021. A tomato NAC transcription factor, SlNAM1, positively regulates ethylene biosynthesis and the onset of tomato fruit ripening. The Plant Journal 108, 1317–1331. 34580960 10.1111/tpj.15512

[eraf357-B43] Gao Y, Lin Y, Xu M, et al., 2022. The role and interaction between transcription factor NAC-NOR and DNA demethylase SlDML2 in the biosynthesis of tomato fruit flavor volatiles. New Phytologist 235, 1913–1926. 35686614 10.1111/nph.18301

[eraf357-B44] Gillaspy G, Ben-David H, Gruissem W. 1993. Fruits: a developmental perspective. The Plant Cell 5, 1439–1451. 12271039 10.1105/tpc.5.10.1439 PMC160374

[eraf357-B45] Gimenez E, Castañeda L, Pineda B, Pan IL, Moreno V, Angosto T, Lozano R. 2016. *TOMATO AGAMOUS1* and *ARLEQUIN/TOMATO AGAMOUS-LIKE1* MADS-box genes have redundant and divergent functions required for tomato reproductive development. Plant Molecular Biology 91, 513–531. 27125648 10.1007/s11103-016-0485-4

[eraf357-B46] Giovannoni J, Tanksley S, Vrebalov J, Noensie F. 2004. NOR gene compositions and methods for use thereof. Patent US 6762347 B1.

[eraf357-B47] Giovannoni JJ . 2004. Genetic regulation of fruit development and ripening. The Plant Cell 16, S170–S180. 15010516 10.1105/tpc.019158 PMC2643394

[eraf357-B48] Guo H, Ecker JR. 2003. Plant responses to ethylene gas are mediated by SCF^EBF1 EBF2^-dependent proteolysis of EIN3 transcription factor. Cell 115, 667–677. 14675532 10.1016/s0092-8674(03)00969-3

[eraf357-B49] Hao Y, Hu G, Breitel D, Liu M, Mila I, Frasse P, Fu Y, Aharoni A, Bouzayen M, Zouine M. 2015. Auxin response factor SlARF2 is an essential component of the regulatory mechanism controlling fruit ripening in tomato. PLoS Genetics 11, e1005649. 26716451 10.1371/journal.pgen.1005649 PMC4696797

[eraf357-B50] He X, Liu K, Wu Y, Xu W, Wang R, Pirrello J, Bouzayen M, Wu M, Liu M. 2024. A transcriptional cascade mediated by two APETALA2 family members orchestrates carotenoid biosynthesis in tomato. Journal of Integrative Plant Biology 66, 1227–1241. 38546046 10.1111/jipb.13650

[eraf357-B51] He Y, Liu H, Li H, Jin M, Wang X, Yin X, Zhu Q, Rao J. 2021. Transcription factors DkBZR1/2 regulate cell wall degradation genes and ethylene biosynthesis genes during persimmon fruit ripening. Journal of Experimental Botany 72, 6437–6446. 34185065 10.1093/jxb/erab312

[eraf357-B52] Heo JB, Lee Y-S, Sung S. 2013. Epigenetic regulation by long noncoding RNAs in plants. Chromosome Research 21, 685–693. 24233054 10.1007/s10577-013-9392-6 PMC4049567

[eraf357-B53] Hu J, Israeli A, Ori N, Sun T. 2018. The interaction between DELLA and ARF/IAA mediates crosstalk between gibberellin and auxin signaling to control fruit initiation in tomato. The Plant Cell 30, 1710–1728. 30008445 10.1105/tpc.18.00363 PMC6139683

[eraf357-B54] Hu J, Li X, Sun T. 2023. Four class A AUXIN RESPONSE FACTORs promote tomato fruit growth despite suppressing fruit set. Nature Plants 9, 706–719. 37037878 10.1038/s41477-023-01396-y PMC10276352

[eraf357-B55] Hu ZL, Deng L, Chen XQ, Wang PQ, Chen GP. 2010. Co-suppression of the *EIN2*-homology gene *LeEIN2* inhibits fruit ripening and reduces ethylene sensitivity in tomato. Russian Journal of Plant Physiology 57, 554–559.

[eraf357-B56] Huang B, Hu G, Wang K, et al 2021. Interaction of two MADS-box genes leads to growth phenotype divergence of all-flesh type of tomatoes. Nature Communications 12, 6892. 10.1038/s41467-021-27117-7 PMC8616914 34824241

[eraf357-B57] Huang W, Xian Z, Kang X, Tang N, Li Z. 2015. Genome-wide identification, phylogeny and expression analysis of GRAS gene family in tomato. BMC Plant Biology 15, 209. 26302743 10.1186/s12870-015-0590-6 PMC4549011

[eraf357-B58] Huang W, Hu N, Xiao Z, Qiu Y, Yang Y, Yang J, Mao X, Wang Y, Li Z, Guo H. 2022. A molecular framework of ethylene-mediated fruit growth and ripening processes in tomato. The Plant Cell 34, 3280–3300. 35604102 10.1093/plcell/koac146 PMC9421474

[eraf357-B59] Huang Z, van der Knaap E. 2011. Tomato *fruit weight 11.3* maps close to *fasciated* on the bottom of chromosome 11. Theoretical and Applied Genetics 123, 465–474. 21541852 10.1007/s00122-011-1599-3

[eraf357-B60] Hyodo H, Terao A, Furukawa J, Sakamoto N, Yurimoto H, Satoh S, Iwai H. 2013. Tissue specific localization of pectin-Ca^2+^ cross-linkages and pectin methyl-esterification during fruit ripening in tomato (*Solanum lycopersicum*). PLoS ONE 8, e78949. 24236073 10.1371/journal.pone.0078949 PMC3827314

[eraf357-B61] Itkin M, Seybold H, Breitel D, Rogachev I, Meir S, Aharoni A. 2009. TOMATO AGAMOUS-LIKE 1 is a component of the fruit ripening regulatory network. The Plant Journal 60, 1081–1095. 19891701 10.1111/j.1365-313X.2009.04064.x

[eraf357-B62] Ito Y, Nishizawa-Yokoi A, Endo M, Mikami M, Shima Y, Nakamura N, Kotake-Nara E, Kawasaki S, Toki S. 2017. Re-evaluation of the *rin* mutation and the role of *RIN* in the induction of tomato ripening. Nature Plants 3, 866–874. 29085071 10.1038/s41477-017-0041-5

[eraf357-B63] Jeena GS, Kumar S, Shukla RK. 2019. Structure, evolution and diverse physiological roles of SWEET sugar transporters in plants. Plant Molecular Biology 100, 351–365. 31030374 10.1007/s11103-019-00872-4

[eraf357-B64] Jia H, Xu Y, Deng Y, Xie Y, Gao Z, Lang Z, Niu Q. 2024. Key transcription factors regulate fruit ripening and metabolite accumulation in tomato. Plant Physiology 195, 2256–2273. 38561990 10.1093/plphys/kiae195 PMC11213253

[eraf357-B65] Jiang X, Lubini G, Hernandes-Lopes J, Rijnsburger K, Veltkamp V, de Maagd RA, Angenent GC, Bemer M. 2022. FRUITFULL-like genes regulate flowering time and inflorescence architecture in tomato. The Plant Cell 34, 1002–1019. 34893888 10.1093/plcell/koab298 PMC8894982

[eraf357-B66] Jones B, Frasse P, Olmos E, Zegzouti H, Li ZG, Latché A, Pech JC, Bouzayen M. 2002. Down-regulation of DR12, an auxin-response-factor homolog, in the tomato results in a pleiotropic phenotype including dark green and blotchy ripening fruit. The Plant Journal 32, 603–613. 12445130 10.1046/j.1365-313x.2002.01450.x

[eraf357-B67] Jones CM . 2003. Characterization and inheritance of the *Anthocyanin fruit* (*Aft*) tomato. Journal of Heredity 94, 449–456. 14691311 10.1093/jhered/esg093

[eraf357-B68] Ju C, Yoon GM, Shemansky JM, et al 2012. CTR1 phosphorylates the central regulator EIN2 to control ethylene hormone signaling from the ER membrane to the nucleus in *Arabidopsis*. Proceedings of the National Academy of Sciences, USA 109, 19486–19491. 10.1073/pnas.1214848109 PMC3511113 23132950

[eraf357-B69] Jumper J, Evans R, Pritzel A, et al 2021. Highly accurate protein structure prediction with AlphaFold. Nature 596, 583–589. 34265844 10.1038/s41586-021-03819-2 PMC8371605

[eraf357-B70] Karlova R, Rosin FM, Busscher-Lange J, Parapunova V, Do PT, Fernie AR, Fraser PD, Baxter C, Angenent GC, de Maagd RA. 2011. Transcriptome and metabolite profiling show that APETALA2a is a major regulator of tomato fruit ripening. The Plant Cell 23, 923–941. 21398570 10.1105/tpc.110.081273 PMC3082273

[eraf357-B71] Karlova R, van Haarst JC, Maliepaard C, van de Geest H, Bovy AG, Lammers M, Angenent GC, de Maagd RA. 2013. Identification of microRNA targets in tomato fruit development using high-throughput sequencing and degradome analysis. Journal of Experimental Botany 64, 1863–1878. 23487304 10.1093/jxb/ert049 PMC3638818

[eraf357-B72] Karlova R, Chapman N, David K, Angenent GC, Seymour GB, de Maagd RA. 2014. Transcriptional control of fleshy fruit development and ripening. Journal of Experimental Botany 65, 4527–4541. 25080453 10.1093/jxb/eru316

[eraf357-B73] Lang Z, Wang Y, Tang K, Tang D, Datsenka T, Cheng J, Zhang Y, Handa AK, Zhu J-K. 2017. Critical roles of DNA demethylation in the activation of ripening-induced genes and inhibition of ripening-repressed genes in tomato fruit. Proceedings of the National Academy of Sciences, USA 114, E4511–E4519. 10.1073/pnas.1705233114 PMC5465898 28507144

[eraf357-B74] Lashbrook CC, Gonzalez-Bosch C, Bennett AB. 1994. Two divergent endo-beta-1,4-glucanase genes exhibit overlapping expression in ripening fruit and abscising flowers. The Plant Cell 6, 1485. 7994180 10.1105/tpc.6.10.1485 PMC160536

[eraf357-B75] Latchman DS . 1997. Transcription factors: an overview. International Journal of Biochemistry & Cell Biology 29, 1305–1312. 9570129 10.1016/s1357-2725(97)00085-x

[eraf357-B76] Latifi-Navid H, Mokhtari S, Taghizadeh S, Moradi F, Poostforoush-Fard D, Alijanpour S, Aghanoori M-R. 2025. AI-assisted multi-OMICS analysis reveals new markers for the prediction of AD. Biochimica et Biophysica Acta (BBA) - Molecular Basis of Disease 1871, 167925. 40441366 10.1016/j.bbadis.2025.167925

[eraf357-B77] Lavy M, Estelle M. 2016. Mechanisms of auxin signaling. Development 143, 3226–3229. 27624827 10.1242/dev.131870 PMC5047657

[eraf357-B78] Lee JM, Joung J, McQuinn R, Chung M, Fei Z, Tieman D, Klee H, Giovannoni J. 2012. Combined transcriptome, genetic diversity and metabolite profiling in tomato fruit reveals that the ethylene response factor *SlERF6* plays an important role in ripening and carotenoid accumulation. The Plant Journal 70, 191–204. 22111515 10.1111/j.1365-313X.2011.04863.x

[eraf357-B79] Leseberg CH, Eissler CL, Wang X, Johns MA, Duvall MR, Mao L. 2008. Interaction study of MADS-domain proteins in tomato. Journal of Experimental Botany 59, 2253–2265. 18487636 10.1093/jxb/ern094

[eraf357-B80] Li F, Fu M, Zhou S, Xie Q, Chen G, Chen X, Hu Z. 2023. A tomato HD-zip I transcription factor, VAHOX1, acts as a negative regulator of fruit ripening. Horticulture Research 10, 1026–1036. 10.1093/hr/uhac236 PMC9832867 36643762

[eraf357-B81] Li N, He Q, Wang J, et al 2023. Super-pangenome analyses highlight genomic diversity and structural variation across wild and cultivated tomato species. Nature Genetics 55, 852–860. 37024581 10.1038/s41588-023-01340-y PMC10181942

[eraf357-B82] Li R, Fu D, Zhu B, Luo Y, Zhu H. 2018. CRISPR/Cas9-mediated mutagenesis of *lncRNA1459* alters tomato fruit ripening. The Plant Journal 94, 513–524. 29446503 10.1111/tpj.13872

[eraf357-B83] Li S, Chen K, Grierson D. 2021. Molecular and hormonal mechanisms regulating fleshy fruit ripening. Cells 10, 1136. 34066675 10.3390/cells10051136 PMC8151651

[eraf357-B84] Li W, Ma M, Feng Y, Li H, Wang Y, Ma Y, Li M, An F, Guo H. 2015. EIN2-directed translational regulation of ethylene signaling in *Arabidopsis*. Cell 163, 670–683. 26496607 10.1016/j.cell.2015.09.037

[eraf357-B85] Li Y, Zhu B, Xu W, Zhu H, Chen A, Xie Y, Shao Y, Luo Y. 2007. *LeERF1* positively modulated ethylene triple response on etiolated seedling, plant development and fruit ripening and softening in tomato. Plant Cell Reports 26, 1999–2008. 17639404 10.1007/s00299-007-0394-8

[eraf357-B86] Li Z, Jiang G, Liu X, et al 2020. Histone demethylase SlJMJ6 promotes fruit ripening by removing H3K27 methylation of ripening-related genes in tomato. New Phytologist 227, 1138–1156. 32255501 10.1111/nph.16590

[eraf357-B87] Lin T, Zhu G, Zhang J, et al 2014. Genomic analyses provide insights into the history of tomato breeding. Nature Genetics 46, 1220–1226. 25305757 10.1038/ng.3117

[eraf357-B88] Lin Z, Hong Y, Yin M, Li C, Zhang K, Grierson D. 2008. A tomato HD-Zip homeobox protein, LeHB-1, plays an important role in floral organogenesis and ripening. The Plant Journal 55, 301–310. 18397374 10.1111/j.1365-313X.2008.03505.x PMC2607530

[eraf357-B89] Liu C, Lu F, Cui X, Cao X. 2010. Histone methylation in higher plants. Annual Review of Plant Biology 61, 395–420. 10.1146/annurev.arplant.043008.091939 20192747

[eraf357-B90] Liu G, Li C, Yu H, et al 2020. *GREEN STRIPE*, encoding methylated TOMATO AGAMOUS-LIKE 1, regulates chloroplast development and Chl synthesis in fruit. New Phytologist 228, 302–317. 32463946 10.1111/nph.16705

[eraf357-B91] Liu H, Liu L, Liang D, et al 2021. SlBES1 promotes tomato fruit softening through transcriptional inhibition of *PMEU1*. iScience 24, 102926. 34430815 10.1016/j.isci.2021.102926 PMC8374504

[eraf357-B92] Liu H, Zhang J, Zhang R, Chen C, Tao J, Xiong J, Xiong A. 2025. SlMYB1R1-*SlSWEET12c* module synergistically promotes sugar accumulation in tomato fruits. The Plant Journal 121, e70062. 39985809 10.1111/tpj.70062

[eraf357-B93] Liu J, Van Eck J, Cong B, Tanksley SD. 2002. A new class of regulatory genes underlying the cause of pear-shaped tomato fruit. Proceedings of the National Academy of Sciences, USA 99, 13302–13306. 10.1073/pnas.162485999 PMC130628 12242331

[eraf357-B94] Liu M, Gomes BL, Mila I, et al 2016. Comprehensive profiling of ethylene response factor expression identifies ripening-associated *ERF* genes and their link to key regulators of fruit ripening in tomato. Plant Physiology 170, 1732–1744. 26739234 10.1104/pp.15.01859 PMC4775140

[eraf357-B95] Liu R, How-Kit A, Stammitti L, et al 2015. A DEMETER-like DNA demethylase governs tomato fruit ripening. Proceedings of the National Academy of Sciences, USA 112, 10804–10809. 10.1073/pnas.1503362112 PMC4553810 26261318

[eraf357-B96] Liu Y, Shi Y, Su D, Lu W, Li Z. 2021. SlGRAS4 accelerates fruit ripening by regulating ethylene biosynthesis genes and SlMADS1 in tomato. Horticulture Research 8, 3. 33384413 10.1038/s41438-020-00431-9 PMC7775462

[eraf357-B97] Livne S, Lor VS, Nir I, Eliaz N, Aharoni A, Olszewski NE, Eshed Y, Weiss D. 2015. Uncovering DELLA-independent gibberellin responses by characterizing new tomato *procera* mutants. The Plant Cell 27, 1579–1594. 26036254 10.1105/tpc.114.132795 PMC4498196

[eraf357-B98] Lü P, Yu S, Zhu N, et al 2018. Genome encode analyses reveal the basis of convergent evolution of fleshy fruit ripening. Nature Plants 4, 784–791. 30250279 10.1038/s41477-018-0249-z

[eraf357-B99] Lye ZN, Purugganan MD. 2019. Copy number variation in domestication. Trends in Plant Science 24, 352–365. 30745056 10.1016/j.tplants.2019.01.003

[eraf357-B100] Machida C, Nakagawa A, Kojima S, Takahashi H, Machida Y. 2015. The complex of ASYMMETRIC LEAVES (AS) proteins plays a central role in antagonistic interactions of genes for leaf polarity specification in *Arabidopsis*. WIREs Developmental Biology 4, 655–671. 26108442 10.1002/wdev.196 PMC4744985

[eraf357-B101] Mapelli S, National I. 1978. Relationship between set, development and activities of growth regulators in tomato fruits. Plant & Cell Physiology 19, 1281–1288.

[eraf357-B102] Martel C, Vrebalov J, Tafelmeyer P, Giovannoni JJ. 2011. The tomato MADS-box transcription factor RIPENING INHIBITOR interacts with promoters involved in numerous ripening processes in a COLORLESS NONRIPENING-dependent manner. Plant Physiology 157, 1568–1579. 21941001 10.1104/pp.111.181107 PMC3252172

[eraf357-B103] Mata CI, Fabre B, Parsons HT, Hertog MLATM, Van Raemdonck G, Baggerman G, Van de Poel B, Lilley KS, Nicolaï BM. 2018. Ethylene receptors, CTRs and EIN2 target protein identification and quantification through parallel reaction monitoring during tomato fruit ripening. Frontiers in Plant Science 9, 1626. 30467512 10.3389/fpls.2018.01626 PMC6235968

[eraf357-B104] Maupilé L, Chaib J, Boualem A, Bendahmane A. 2024. Parthenocarpy, a pollination-independent fruit set mechanism to ensure yield stability. Trends in Plant Science 29, 1254–1265. 39034223 10.1016/j.tplants.2024.06.007

[eraf357-B105] Meng F, Liu H, Hu S, et al 2023. The brassinosteroid signaling component SlBZR1 promotes tomato fruit ripening and carotenoid accumulation. Journal of Integrative Plant Biology 65, 1794–1813. 37009849 10.1111/jipb.13491

[eraf357-B106] Meng L, Fan Z, Zhang Q, et al 2018. *BEL 1-LIKE HOMEODOMAIN 11* regulates chloroplast development and chlorophyll synthesis in tomato fruit. The Plant Journal 94, 1126–1140. 29659108 10.1111/tpj.13924

[eraf357-B107] Merchante C, Brumos J, Yun J, Hu Q, Spencer KR, Enríquez P, Binder BM, Heber S, Stepanova AN, Alonso JM. 2015. Gene-specific translation regulation mediated by the hormone-signaling molecule EIN2. Cell 163, 684–697. 26496608 10.1016/j.cell.2015.09.036

[eraf357-B108] Mou W, Li D, Luo Z, Li L, Mao L, Ying T. 2018. SlAREB1 transcriptional activation of *NOR* is involved in abscisic acid-modulated ethylene biosynthesis during tomato fruit ripening. Plant Science 276, 239–249. 30348324 10.1016/j.plantsci.2018.07.015

[eraf357-B109] Moxon S, Jing R, Szittya G, Schwach F, Rusholme Pilcher RL, Moulton V, Dalmay T. 2008. Deep sequencing of tomato short RNAs identifies microRNAs targeting genes involved in fruit ripening. Genome Research 18, 1602–1609. 18653800 10.1101/gr.080127.108 PMC2556272

[eraf357-B110] Muños S, Ranc N, Botton E, et al 2011. Increase in tomato locule number is controlled by two single-nucleotide polymorphisms located near *WUSCHEL*. Plant Physiology 156, 2244–2254. 21673133 10.1104/pp.111.173997 PMC3149950

[eraf357-B111] Nadakuduti SS, Holdsworth WL, Klein CL, Barry CS. 2014. *KNOX* genes influence a gradient of fruit chloroplast development through regulation of *GOLDEN2-LIKE* expression in tomato. The Plant Journal 78, 1022–1033. 24689783 10.1111/tpj.12529

[eraf357-B112] Nguyen CV, Vrebalov JT, Gapper NE, Zheng Y, Zhong S, Fei Z, Giovannoni JJ. 2014. Tomato *GOLDEN2-LIKE* transcription factors reveal molecular gradients that function during fruit development and ripening. The Plant Cell 26, 585–601. 24510723 10.1105/tpc.113.118794 PMC3967027

[eraf357-B113] Niu Q, Xu Y, Huang H, et al 2025. Two transcription factors play critical roles in mediating epigenetic regulation of fruit ripening in tomato. Proceedings of the National Academy of Sciences, USA 122, 2017. 10.1073/pnas.2422798122 PMC12012504 40203043

[eraf357-B114] Niu X, Li H, Li R, et al 2022. Transcription factor SlBEL2 interferes with GOLDEN2-LIKE and influences green shoulder formation in tomato fruits. The Plant Journal 112, 982–997. 36164829 10.1111/tpj.15989

[eraf357-B115] Pan IL, McQuinn R, Giovannoni JJ, Irish VF. 2010. Functional diversification of *AGAMOUS* lineage genes in regulating tomato flower and fruit development. Journal of Experimental Botany 61, 1795–1806. 20335407 10.1093/jxb/erq046 PMC2852668

[eraf357-B116] Peng Z, Liu G, Li H, Wang Y, Gao H, Jemrić T, Fu D. 2022. Molecular and genetic events determining the softening of fleshy fruits: a comprehensive review. International Journal of Molecular Sciences 23, 12482. 36293335 10.3390/ijms232012482 PMC9604029

[eraf357-B117] Pnueli L, Hareven D, Rounsley SD, Yanofsky MF, Lifschitz E. 1994. Isolation of the tomato *AGAMOUS* gene *TAG1* and analysis of its homeotic role in transgenic plants. The Plant Cell 6, 163–173. 7908549 10.1105/tpc.6.2.163 PMC160424

[eraf357-B118] Potuschak T, Lechner E, Parmentier Y, Yanagisawa S, Grava S, Koncz C, Genschik P. 2003. EIN3-dependent regulation of plant ethylene hormone signaling by two *Arabidopsis* F box proteins: EBF1 and EBF2. Cell 115, 679–689. 14675533 10.1016/s0092-8674(03)00968-1

[eraf357-B119] Powell ALT, Nguyen C V, Hill T, et al 2012. *Uniform ripening* encodes a *Golden 2-like* transcription factor regulating tomato fruit chloroplast development. Science 336, 1711–1715. 22745430 10.1126/science.1222218

[eraf357-B120] Qiao H, Chang KN, Yazaki J, Ecker JR. 2009. Interplay between ethylene, ETP1/ETP2 F-box proteins, and degradation of EIN2 triggers ethylene responses in *Arabidopsis*. Genes & Development 23, 512–521. 19196655 10.1101/gad.1765709 PMC2648649

[eraf357-B121] Qiao H, Shen Z, Huang SC, Schmitz RJ, Urich MA, Briggs SP, Ecker JR. 2012. Processing and subcellular trafficking of ER-tethered EIN2 control response to ethylene gas. Science 338, 390–393. 22936567 10.1126/science.1225974 PMC3523706

[eraf357-B122] Riechmann JL, Krizek BA, Meyerowitz EM. 1996. Dimerization specificity of *Arabidopsis* MADS domain homeotic proteins APETALA1, APETALA3, PISTILLATA, and AGAMOUS. Proceedings of the National Academy of Sciences, USA 93, 4793–4798. 10.1073/pnas.93.10.4793 PMC39358 8643482

[eraf357-B123] Robinson RW, Tomes ML. 1968. Ripening inhibitor: a gene with multiple effect on ripening. Report of the Tomato Genetics Cooperative 18, 36–37.

[eraf357-B124] Rodríguez-Leal D, Lemmon ZH, Man J, Bartlett ME, Lippman ZB. 2017. Engineering quantitative trait variation for crop improvement by genome editing. Cell 171, 470–480. 28919077 10.1016/j.cell.2017.08.030

[eraf357-B125] Ronen G, Carmel-Goren L, Zamir D, Hirschberg J. 2000. An alternative pathway to *β*-carotene formation in plant chromoplasts discovered by map-based cloning of *Beta* and *old-gold* color mutations in tomato. Proceedings of the National Academy of Sciences, USA 97, 11102–11107. 10.1073/pnas.190177497 PMC27155 10995464

[eraf357-B126] Sagar M, Chervin C, Mila I, et al 2013. SlARF4, an auxin response factor involved in the control of sugar metabolism during tomato fruit development. Plant Physiology 161, 1362–1374. 23341361 10.1104/pp.113.213843 PMC3585602

[eraf357-B127] Sang K, Li J, Qian X, Yu J, Zhou Y, Xia X. 2022. The APETALA2a/DWARF/BRASSINAZOLE-RESISTANT 1 module contributes to carotenoid synthesis in tomato fruits. The Plant Journal 112, 1238–1251. 36271694 10.1111/tpj.16009

[eraf357-B128] Sato S, Tabata S, Hirakawa H, et al 2012. The tomato genome sequence provides insights into fleshy fruit evolution. Nature 485, 635–641. 22660326 10.1038/nature11119 PMC3378239

[eraf357-B129] Shen H, Zhou Y, Xiao H, Ding Y, Chen G, Yang Z, Hu Z, Wu T. 2024. SlFSR positively regulates ethylene biosynthesis and lycopene accumulation during fruit ripening in tomato. Plant Physiology and Biochemistry 215, 109008. 39226760 10.1016/j.plaphy.2024.109008

[eraf357-B130] Shi Y, Vrebalov J, Zheng H, et al 2021. A tomato LATERAL ORGAN BOUNDARIES transcription factor, *SlLOB1*, predominantly regulates cell wall and softening components of ripening. Proceedings of the National Academy of Sciences, USA 118, e2102486118. 10.1073/pnas.2102486118 PMC8379924 34380735

[eraf357-B131] Shima Y, Kitagawa M, Fujisawa M, Nakano T, Kato H, Kimbara J, Kasumi T, Ito Y. 2013. Tomato FRUITFULL homologues act in fruit ripening via forming MADS-box transcription factor complexes with RIN. Plant Molecular Biology 82, 427–438. 23677393 10.1007/s11103-013-0071-y

[eraf357-B132] Shin J-H, Mila I, Liu M, Rodrigues MA, Vernoux T, Pirrello J, Bouzayen M. 2019. The RIN-regulated Small Auxin-Up RNA SAUR 69 is involved in the unripe-to-ripe phase transition of tomato fruit via enhancement of the sensitivity to ethylene. New Phytologist 222, 820–836. 30511456 10.1111/nph.15618

[eraf357-B133] Shinozaki Y, Nicolas P, Fernandez-Pozo N, et al 2018. High-resolution spatiotemporal transcriptome mapping of tomato fruit development and ripening. Nature Communications 9, 364. 10.1038/s41467-017-02782-9 PMC5785480 29371663

[eraf357-B134] Song J, Shang L, Li C, et al 2022. Variation in the fruit development gene *POINTED TIP* regulates protuberance of tomato fruit tip. Nature Communications 13, 5940. 10.1038/s41467-022-33648-4 PMC9547884 36209204

[eraf357-B135] Su G, Lin Y, Wang C, et al 2023. Expansin SlExp1 and endoglucanase SlCel2 synergistically promote fruit softening and cell wall disassembly in tomato. The Plant Cell 36, 709–726. 10.1093/plcell/koad291 PMC10896287 38000892

[eraf357-B136] Su X, Wang B, Geng X, et al 2021. A high-continuity and annotated tomato reference genome. BMC Genomics 22, 898. 34911432 10.1186/s12864-021-08212-x PMC8672587

[eraf357-B137] Sun S, Yuan Y, Xu M, Liu Z, Yuan X, Li X, Li R, Cui X. 2025. TKN3 affects cell expansion to regulate fruit development in tomato. Horticultural Plant Journal 11, 251–263.

[eraf357-B138] Sun Y, Xiao H. 2015. Identification of alternative splicing events by RNA sequencing in early growth tomato fruits. BMC Genomics 16, 948. 26573826 10.1186/s12864-015-2128-6 PMC4647595

[eraf357-B139] Swinnen G, Goossens A, Pauwels L. 2016. Lessons from domestication: targeting *cis*-regulatory elements for crop improvement. Trends in Plant Science 21, 506–515. 26876195 10.1016/j.tplants.2016.01.014

[eraf357-B140] Szemenyei H, Hannon M, Long JA. 2008. TOPLESS mediates auxin-dependent transcriptional repression during *Arabidopsis* embryogenesis. Science 319, 1384–1386. 18258861 10.1126/science.1151461

[eraf357-B141] Szymański J, Bocobza S, Panda S, et al 2020. Analysis of wild tomato introgression lines elucidates the genetic basis of transcriptome and metabolome variation underlying fruit traits and pathogen response. Nature Genetics 52, 1111–1121. 32989321 10.1038/s41588-020-0690-6

[eraf357-B142] Takei H, Shirasawa K, Kuwabara K, Toyoda A, Matsuzawa Y, Iioka S, Ariizumi T. 2021. *De novo* genome assembly of two tomato ancestors, *Solanum pimpinellifolium* and *Solanum lycopersicum* var. *cerasiforme*, by long-read sequencing. DNA Research 28, dsaa029. 33475141 10.1093/dnares/dsaa029 PMC7934570

[eraf357-B143] Tang D, Gallusci P, Lang Z. 2020. Fruit development and epigenetic modifications. New Phytologist 228, 839–844. 32506476 10.1111/nph.16724

[eraf357-B144] Tang X, Zhang Y. 2023. Beyond knockouts: fine-tuning regulation of gene expression in plants with CRISPR-Cas-based promoter editing. New Phytologist 239, 868–874. 37282668 10.1111/nph.19020

[eraf357-B145] Tanksley SD . 2004. The genetic, developmental, and molecular bases of fruit size and shape variation in tomato. The Plant Cell 16, S181–S189. 15131251 10.1105/tpc.018119 PMC2643388

[eraf357-B146] Teyssier E, Bernacchia G, Maury S, How Kit A, Stammitti-Bert L, Rolin D, Gallusci P. 2008. Tissue dependent variations of DNA methylation and endoreduplication levels during tomato fruit development and ripening. Planta 228, 391–399. 18488247 10.1007/s00425-008-0743-z

[eraf357-B147] Theißen G, Saedler H. 2001. Floral quartets. Nature 409, 469–471. 10.1038/35054172 11206529

[eraf357-B148] Thompson AJ, Tor M, Barry CS, Vrebalov J, Orfila C, Jarvis MC, Giovannoni JJ, Grierson D, Seymour GB. 1999. Molecular and genetic characterization of a novel pleiotropic tomato-ripening mutant. Plant Physiology 120, 383–390. 10364389 10.1104/pp.120.2.383 PMC59276

[eraf357-B149] Tieman DM, Taylor MG, Ciardi JA, Klee HJ. 2000. The tomato ethylene receptors NR and LeETR4 are negative regulators of ethylene response and exhibit functional compensation within a multigene family. Proceedings of the National Academy of Sciences, USA 97, 5663–5668. 10.1073/pnas.090550597 PMC25885 10792050

[eraf357-B150] Tigchelaar E, Tomes M, Kerr E, Barman R. 1973. A new fruit ripening mutant, *non-ripening* (*nor*). Report of the Tomato Genetics Cooperative 23, 33–34.

[eraf357-B151] Uluisik S, Chapman NH, Smith R, et al 2016. Genetic improvement of tomato by targeted control of fruit softening. Nature Biotechnology 34, 950–952. 10.1038/nbt.3602 27454737

[eraf357-B152] Van de Poel B, Vandenzavel N, Smet C, et al 2014. Tissue specific analysis reveals a differential organization and regulation of both ethylene biosynthesis and E8 during climacteric ripening of tomato. BMC Plant Biology 14, 11. 24401128 10.1186/1471-2229-14-11 PMC3900696

[eraf357-B153] Varga A, Bruinsma J. 1976. Roles of seeds and auxins in tomato fruit growth. Zeitschrift für Pflanzenphysiologie 80, 95–104.

[eraf357-B154] Vrebalov J, Ruezinsky D, Padmanabhan V, White R, Medrano D, Drake R, Schuch W, Giovannoni J. 2002. A MADS-box gene necessary for fruit ripening at the tomato *ripening-inhibitor* (*rin*) locus. Science 296, 343–346. 11951045 10.1126/science.1068181

[eraf357-B155] Vrebalov J, Pan IL, Arroyo AJM, et al 2009. Fleshy fruit expansion and ripening are regulated by the tomato *SHATTERPROOF* gene *TAGL1*. The Plant Cell 21, 3041–3062. 19880793 10.1105/tpc.109.066936 PMC2782289

[eraf357-B156] Wang D, Seymour GB. 2022. Molecular and biochemical basis of softening in tomato. Molecular Horticulture 2, 5. 37789493 10.1186/s43897-022-00026-z PMC10515243

[eraf357-B157] Wang L, Koppitch K, Cutting A, Dong P, Kudtarkar P, Zeng J, Cameron RA, Davidson EH. 2019. Developmental effector gene regulation: multiplexed strategies for functional analysis. Developmental Biology 445, 68–79. 30392838 10.1016/j.ydbio.2018.10.018 PMC6321769

[eraf357-B158] Wang R, Tavano ECR, Lammers M, Martinelli AP, Angenent GC, de Maagd RA. 2019. Re-evaluation of transcription factor function in tomato fruit development and ripening with CRISPR/Cas9-mutagenesis. Scientific Reports 9, 1696. 30737425 10.1038/s41598-018-38170-6 PMC6368595

[eraf357-B159] Wang R, Lammers M, Tikunov Y, Bovy AG, Angenent GC, de Maagd RA. 2020. The *rin*, *nor* and *Cnr* spontaneous mutations inhibit tomato fruit ripening in additive and epistatic manners. Plant Science 294, 110436. 32234221 10.1016/j.plantsci.2020.110436

[eraf357-B160] Wang R, Liu K, Tang B, Su D, He X, Deng H, Wu M, Bouzayen M, Grierson D, Liu M. 2023. The MADS-box protein SlTAGL1 regulates a ripening-associated *SlDQD/SDH2* involved in flavonoid biosynthesis and resistance against *Botrytis cinerea* in post-harvest tomato fruit. The Plant Journal 115, 1746–1757. 37326247 10.1111/tpj.16354

[eraf357-B161] Wang X, Gao L, Jiao C, et al 2020. Genome of *Solanum pimpinellifolium* provides insights into structural variants during tomato breeding. Nature Communications 11, 5817. 10.1038/s41467-020-19682-0 PMC7670462 33199703

[eraf357-B162] Weijers D, Wagner D. 2016. Transcriptional responses to the auxin hormone. Annual Review of Plant Biology 67, 539–574. 10.1146/annurev-arplant-043015-112122 26905654

[eraf357-B163] Wen X, Zhang C, Ji Y, Zhao Q, He W, An F, Jiang L, Guo H. 2012. Activation of ethylene signaling is mediated by nuclear translocation of the cleaved EIN2 carboxyl terminus. Cell Research 22, 1613–1616. 23070300 10.1038/cr.2012.145 PMC3494400

[eraf357-B164] Wittkopp PJ, Kalay G. 2011. *Cis*-regulatory elements: molecular mechanisms and evolutionary processes underlying divergence. Nature Reviews: Genetics 13, 59–69. 10.1038/nrg3095 22143240

[eraf357-B165] Xiao H, Jiang N, Schaffner E, Stockinger EJ, van der Knaap E. 2008. A retrotransposon-mediated gene duplication underlies morphological variation of tomato fruit. Science 319, 1527–1530. 18339939 10.1126/science.1153040

[eraf357-B166] Xu C, Liberatore KL, Macalister CA, et al 2015. A cascade of arabinosyltransferases controls shoot meristem size in tomato. Nature Genetics 47, 784–792. 26005869 10.1038/ng.3309

[eraf357-B167] Yang T, Ali M, Lin L, et al 2023. Recoloring tomato fruit by CRISPR/Cas9-mediated multiplex gene editing. Horticulture Research 10, uhac214. 36643741 10.1093/hr/uhac214 PMC9832834

[eraf357-B168] Yao Q, Shen R, Shao Y, Tian Y, Han P, Zhang X, Zhu J, Lu Y. 2024. Efficient and multiplex gene upregulation in plants through CRISPR-Cas-mediated knockin of enhancers. Molecular Plant 17, 1472–1483. 39049493 10.1016/j.molp.2024.07.009

[eraf357-B169] Ye J, Wang X, Hu T, et al 2017. An InDel in the promoter of *AI-ACTIVATED MALATE TRANSPORTER9* selected during tomato domestication determines fruit malate contents and aluminum tolerance. The Plant Cell 29, 2249–2268. 28814642 10.1105/tpc.17.00211 PMC5635988

[eraf357-B170] You S, Wu Y, Li W, Liu X, Tang Q, Huang F, Li Y, Wang H, Liu M, Zhang Y. 2024. SlERF.G3-Like mediates a hierarchical transcriptional cascade to regulate ripening and metabolic changes in tomato fruit. Plant Biotechnology Journal 22, 165–180. 37750661 10.1111/pbi.14177 PMC10754011

[eraf357-B171] Yu T, Tzeng DTW, Li R, Chen J, Zhong S, Fu D, Zhu B, Luo Y, Zhu H. 2019. Genome-wide identification of long non-coding RNA targets of the tomato MADS box transcription factor RIN and function analysis. Annals of Botany 123, 469–482. 30376036 10.1093/aob/mcy178 PMC6377105

[eraf357-B172] Yu T, Ai G, Xie Q, et al 2022. Regulation of tomato fruit elongation by transcription factor BZR1.7 through promotion of *SUN* gene expression. Horticulture Research 9, uhac121. 35937861 10.1093/hr/uhac121 PMC9347012

[eraf357-B173] Yuan Y, Mei L, Wu M, et al 2018. SlARF10, an auxin response factor, is involved in chlorophyll and sugar accumulation during tomato fruit development. Journal of Experimental Botany 69, 5507–5518. 30219898 10.1093/jxb/ery328 PMC6255703

[eraf357-B174] Yuan Y, Xu X, Gong Z, et al 2019. Auxin response factor 6A regulates photosynthesis, sugar accumulation, and fruit development in tomato. Horticulture Research 6, 85. 31645946 10.1038/s41438-019-0167-x PMC6804849

[eraf357-B175] Yuste-Lisbona FJ, Fernández-Lozano A, Pineda B, et al 2020. *ENO* regulates tomato fruit size through the floral meristem development network. Proceedings of the National Academy of Sciences, USA 117, 8187–8195. 10.1073/pnas.1913688117 PMC7148573 32179669

[eraf357-B176] Zhang F, Wang L, Qi B, Zhao B, Ko EE, Riggan ND, Chin K, Qiao H. 2017. EIN2 mediates direct regulation of histone acetylation in the ethylene response. Proceedings of the National Academy of Sciences, USA 114, 10274–10279. 10.1073/pnas.1707937114 PMC5617289 28874528

[eraf357-B177] Zhang H, Lang Z, Zhu J-K. 2018. Dynamics and function of DNA methylation in plants. Nature Reviews: Molecular Cell Biology 19, 489–506. 29784956 10.1038/s41580-018-0016-z

[eraf357-B178] Zhang L, Zhu M, Ren L, Li A, Chen G, Hu Z. 2018. The *SlFSR* gene controls fruit shelf-life in tomato. Journal Experimental Botany 69, 2897–2909. 10.1093/jxb/ery116 PMC5972576 29635354

[eraf357-B179] Zhang L, Chen L, Pang S, Zheng Q, Quan S, Liu Y, Xu T, Liu Y, Qi M. 2022. Function analysis of the ERF and DREB subfamilies in tomato fruit development and ripening. Frontiers in Plant Science 13, 849048. 35310671 10.3389/fpls.2022.849048 PMC8931701

[eraf357-B180] Zhang M, Kimatu JN, Xu K, Liu B. 2010. DNA cytosine methylation in plant development. Journal of Genetics and Genomics 37, 1–12. 20171573 10.1016/S1673-8527(09)60020-5

[eraf357-B181] Zhang S, Xu M, Qiu Z, Wang K, Du Y, Gu L, Cui X. 2016. Spatiotemporal transcriptome provides insights into early fruit development of tomato (*Solanum lycopersicum*). Scientific Reports 6, 23173. 26988970 10.1038/srep23173 PMC4796798

[eraf357-B182] Zhang X, Yan F, Tang Y, Yuan Y, Deng W, Li Z. 2015. Auxin response gene *SlARF3* plays multiple roles in tomato development and is involved in the formation of epidermal cells and trichomes. Plant & Cell Physiology 56, 2110–2124. 26412778 10.1093/pcp/pcv136

[eraf357-B183] Zhang X, Feng C, Wang M, Li T, Liu X, Jiang J. 2021. Plasma membrane-localized SlSWEET7a and SlSWEET14 regulate sugar transport and storage in tomato fruits. Horticulture Research 8, 186. 34333539 10.1038/s41438-021-00624-w PMC8325691

[eraf357-B184] Zhao W, Li Y, Fan S, Wen T, Wang M, Zhang L, Zhao L. 2021. The transcription factor WRKY32 affects tomato fruit colour by regulating *YELLOW FRUITED-TOMATO 1*, a core component of ethylene signal transduction. Journal of Experimental Botany 72, 4269–4282. 33773493 10.1093/jxb/erab113

[eraf357-B185] Zhou L, Tian S, Qin G. 2019. RNA methylomes reveal the m^6^A-mediated regulation of DNA demethylase gene *SlDML2* in tomato fruit ripening. Genome Biology 20, 156. 31387610 10.1186/s13059-019-1771-7 PMC6683476

[eraf357-B186] Zhou L, Sun Z, Hu T, et al 2024. Increasing flavonoid contents of tomato fruits through disruption of the SlSPL-CNR, a suppressor of SlMYB12 transcription activity. Plant Biotechnology Journal 22, 290–292. 37902173 10.1111/pbi.14214 PMC10826974

[eraf357-B187] Zhu G, Li R, Zhang L, et al 2024. RNA-protein interactions reveals the pivotal role of lncRNA1840 in tomato fruit maturation. The Plant Journal 120, 526–539. 39226395 10.1111/tpj.16998

[eraf357-B188] Zhu M, Chen G, Zhou S, Tu Y, Wang Y, Dong T, Hu Z. 2014. A new tomato NAC (NAM ATAF1/2/CUC2) transcription factor, SlNAC4, functions as a positive regulator of fruit ripening and carotenoid accumulation. Plant & Cell Physiology 55, 119–135. 24265273 10.1093/pcp/pct162

[eraf357-B189] Zhu Q, Deng L, Chen J, et al 2023. Redesigning the tomato fruit shape for mechanized production. Nature Plants 9, 1659–1674. 37723204 10.1038/s41477-023-01522-w

